# Estrogen deficiency and risk of hearing loss in pediatric Turner syndrome

**DOI:** 10.1172/JCI197932

**Published:** 2026-03-03

**Authors:** Yan Huang, Liyang Liang, Yanfang Ye, Lina Zhang, Li Ling, Zhe Meng, Wei Liu, Jia Guo, Zulin Liu, Zhen Zhao, Zhigang Zhang, Yu Si

**Affiliations:** 1 Department of Otorhinolaryngology,; 2 Institute of Hearing and Speech, and; 3 Department of Pediatrics, Sun Yat-sen Memorial Hospital, Sun Yat-sen University, Guangzhou, China.; 4 Clinical Research Design Division, Clinical Research Centre of Sun Yat-sen Memorial Hospital, Sun Yat-sen University, Guangzhou, China.; 5 Department of Medical Statistics, School of Public Health, Sun Yat-sen University, Guangzhou, China.

**Keywords:** Endocrinology, Neuroscience, Otology, Clinical trials, Sex hormones

## Abstract

**BACKGROUND:**

Estrogen deficiency and progressive hearing loss (HL) are significant concerns in individuals with Turner syndrome (TS). However, whether childhood estrogen deficiency increases HL risk and whether estrogen replacement therapy (ERT) prevents hearing deterioration are still unclear.

**METHODS:**

This prospective cohort study recruited children with TS from a tertiary referral center between 2016 and 2024. All participants received standardized recombinant human growth hormone therapy. Longitudinal monitoring data of hormone levels, metabolic parameters, and annual audiological examinations were recorded. The primary analysis used a multivariate Cox model to estimate the adjusted hazard ratio (HR) of hearing loss between estrogen-deficient and estrogen-normal TS patients without prior exogenous estrogen exposure. The secondary analysis compared annual pure tone average (PTA) and its changes between the ERT and non-ERT groups in a substudy.

**RESULTS:**

Among 87 prepubertal pediatric patients with TS, 48 (55.2%) were estrogen deficient, and 38 HL events occurred over a 35-month median follow-up. The estrogen-deficient group had higher HL incidence (27 cases, 56.3%; 20.6 per 100 person-years [PY]) versus estrogen-normal (11 cases, 28.2%; 8.6 per 100 PY), with estrogen deficiency independently increasing HL risk (HR 2.93, 95% CI 1.21–7.12). Notably, estrogen deficiency also independently predicted abnormal distortion product otoacoustic emissions with an even higher effect size (HR 3.98, 95% CI 1.35–11.76). The substudy found that initiating ERT at the age of 12 significantly preserve auditory function, with the ERT group showing markedly lower PTA and slower hearing deterioration (–1.24 dB/year vs. 1.13 dB/year right ear; –1.85 dB/year vs. 1.04 dB/year left ear, *P* = 0.001).

**CONCLUSION:**

Childhood estrogen deficiency is a modifiable risk factor. Initiating ERT around early adolescence may help hearing preservation.

**TRIAL REGISTRATION:**

Chinese Clinical Trial Registry: ChiCTR2300068063.

**FUNDING:**

National Natural Science Foundation of China (grants 82173154 and 82471155), Fundamental Research Funds for the Central Universities Clinical Research 5010 Program (Sun Yat-sen University, no. 2024004).

## Abstract 2

<strong>Estrogen Deficiency and Risk of Hearing Loss in Pediatric Turner Syndrome</strong>

## Graphical abstract

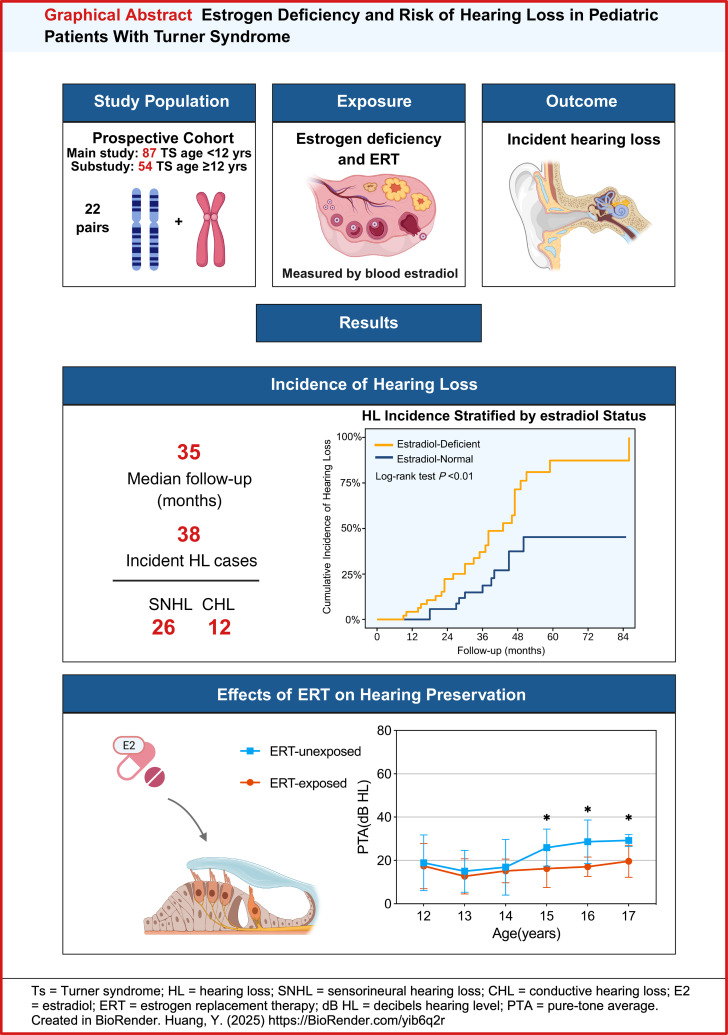

## Introduction

Turner syndrome (TS) is caused by partial or complete X chromosome monosomy, affecting approximately 1 in 2500 to 1 in 4000 live-born female infants (1). It presents with a range of clinical manifestations, including short stature, ovarian dysgenesis, and neurocognitive issues (2). Earlier studies have indicated hearing loss (HL) as a major concern for patients with TS as they age into adolescence and adulthood (3). The Danish nationwide cohort study revealed that women with TS face dramatically elevated incidence rate ratios (IRRs) for otologic pathologies: a 77-fold overall increased risk for middle ear and mastoid diseases (IRR 77, 95% CI 54–108), with particularly striking increases for otitis media (IRR = 56.9), cholesteatoma (IRR = 33.7), and tympanic membrane perforation (IRR = 18.0) relative to healthy women (4). An auditory evaluation of 200 patients with TS with an average age of 27.9 years old found that the annual decrease rate of 8000 Hz high-frequency HL was approximately 1 dB/year. This decline is significantly faster than the age-related HL observed in healthy people (0.6–0.8 dB/year) (5).

Ovarian failure is one of the most prominent clinical characteristics of patients with TS (6). Indirect evidence shows that estrogen deficiency in patients with TS begins in infancy, which is supported by increased gonadotropin levels and delayed bone maturity (7). However, this deficiency is not absolute. It is worth noting that some patients still retain some ovarian functions across the lifespan. This pattern is particularly evident in patients with mosaic karyotypes or structural X-chromosome aberrations. Some of these individuals exhibit preserved estradiol (E_2_) levels that approach age-specific reference ranges during childhood (8). The important point is that some untreated adult women can also maintain their normal E_2_ levels within the range appropriate for their age (9). These findings collectively indicate that not all patients with TS have complete and lifelong estrogen deficiency.

Several studies based on premenopausal and postmenopausal populations showed that there was an independent correlation between estrogen deficiency and the incidence of HL (10–14). However, investigating this relationship in TS requires careful consideration because patients usually begin to receive ERT in adolescence. The use of exogenous hormones in adolescence may confuse the effects of endogenous estrogen deficiency on hearing (6). In order to eliminate this confusion, we investigated patients with TS who had not received estrogen treatment to clarify the undisturbed relationship between endogenous estrogen deficiency and HL. In a cross-sectional study comprising 138 adult patients with TS, a delay in ERT initiation (≥13 years) was associated with an elevated relative risk (RR) for developing conductive HL (CHL) or sensorineural HL (SNHL). However, these associations were not statistically significant and were confounded by age (15). Another cross-sectional study confirmed that women with TS who did not receive ERT in adolescence showed mild sound localization deficits in middle age (16).

Given the existing evidence linking estrogen deficiency to HL and the growing interest in ERT, further research is warranted to clarify the relationship between estrogen deficiency (measured by E_2_ levels) and the incidence of HL in patients with TS. Such findings may support the potential role for estrogen supplementation in reducing the risk of HL in this high-risk population.

The objective of this study was to prospectively examine the association between estrogen deficiency and the incidence of HL in pediatric patients with TS through long-term follow-up of a real-world cohort, and to assess whether initiation of ERT during early adolescence can help preserve auditory function in these individuals.

## Results

### Description of the cohort.

**Figure 1:**
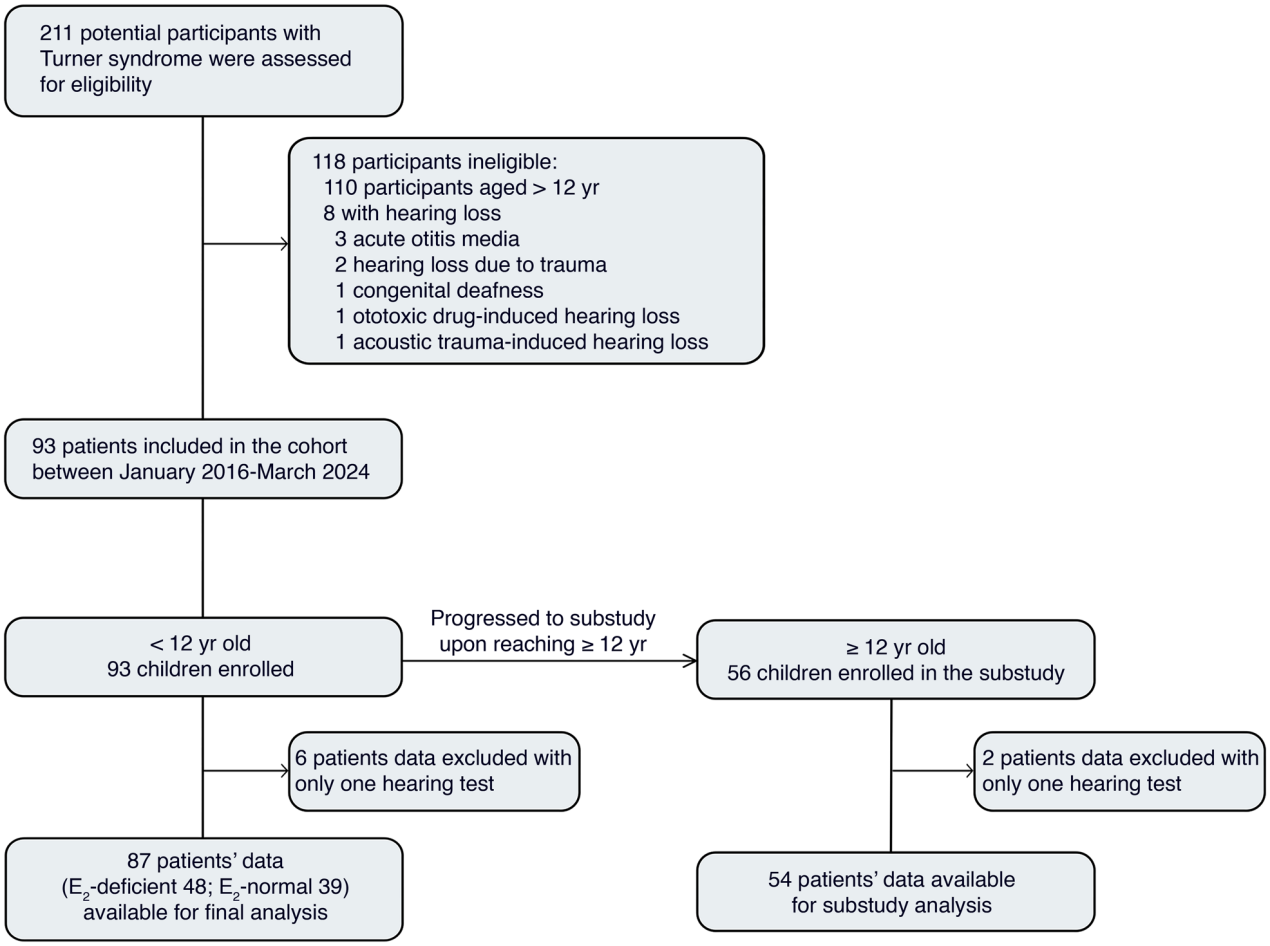
Flowchart of study enrollment. The ChiCTR2300068063 cohort is an ongoing cohort study, with continuous follow-up, and without a prespecified end date. The number of patients included in the cohort between January, 2016 (start of the study) and March, 2024 is presented. The “<12 Years Old” cohort (*n* = 87) was stratified into E_2_-deficient (*n* = 48) and E_2_-normal (*n* = 39) groups to evaluate the association between endogenous E_2_ status and hearing loss. The “≥12 Years Old” substudy cohort (*n* = 54) was used to analyze the impact of initiating exogenous E_2_ replacement therapy on hearing thresholds. E_2_, estradiol.

A total of 211 potential participants with TS were initially assessed for eligibility between January, 2016 and March, 2024. After excluding 118 ineligible individuals (110 due to age >12 years and 8 with preexisting HL), 93 patients under 12 years of age were enrolled in the main prospective cohort. Following the study design, as participants reached 12 years of age, they entered the ERT-focused substudy. To date, 56 children (60.2%) from the original cohort have progressed into this follow-up substudy for longitudinal evaluation of hearing thresholds under exogenous ERT. After excluding 6 patients from the main cohort and 2 from the substudy cohort due to insufficient follow-up data (only 1 hearing test), longitudinal data from 87 participants were available for the final analysis of HL risk in the main study, while 54 participants were included in the substudy analysis of ERT (Figure 1).

To ensure transparent reporting, this study adhered to the STROBE guidelines (17). The median age of children in the main cohort was 8 years (IQR 7.00–10.00). Based on CALIPER-defined thresholds (see Methods) and longitudinal profiling, 87 children with TS were stratified into an E_2_-deficient group (*n* = 48; mean E_2_ = 20.20 pmol/L, 95% CI 18.06–22.34) and an E_2_-normal group (*n* = 39; mean E_2_ = 86.11 pmol/L, 95% CI 71.39–100.82; *P* < 0.001, Supplemental Figure 1; supplemental material available online with this article; https://doi.org/10.1172/JCI197932DS1). The entire cohort exhibited heterogeneous karyotypes (45,X, 31.0%; mosaicism or structural variants, 69.0%) and a high prevalence of systemic comorbidities, including scoliosis (39.1%), renal dysfunction (31.0%), and thyroid disorders (24.1%). A key finding is that the E_2_-deficient subgroup and the E_2_-normal subgroup did not show statistically significant differences in demographic characteristics, karyotype, bone age, or prevalence of major systemic complications (including cardiovascular, metabolic, thyroid, and kidney diseases) (all *P* > 0.05). The primary divergence between the groups was confined to markers of gonadal function; the E_2_-deficient subgroup had significantly smaller ovarian volume (0.57 vs. 1.26 mL, *P* = 0.005), smaller uterine volume (1.61 vs. 4.53 mL, *P* = 0.029), and lower testosterone levels (0.34 vs. 0.55 nmol/L, *P* = 0.001), which is consistent with a phenotype of more severe primary ovarian insufficiency. This indicates that, aside from the expected gonadal function differences, the 2 groups were well balanced in terms of karyotype and major systemic comorbidities at baseline (Table 1 and Supplemental Table 1).

### Association between estrogen deficiency and incident HL.

**Figure 2:**
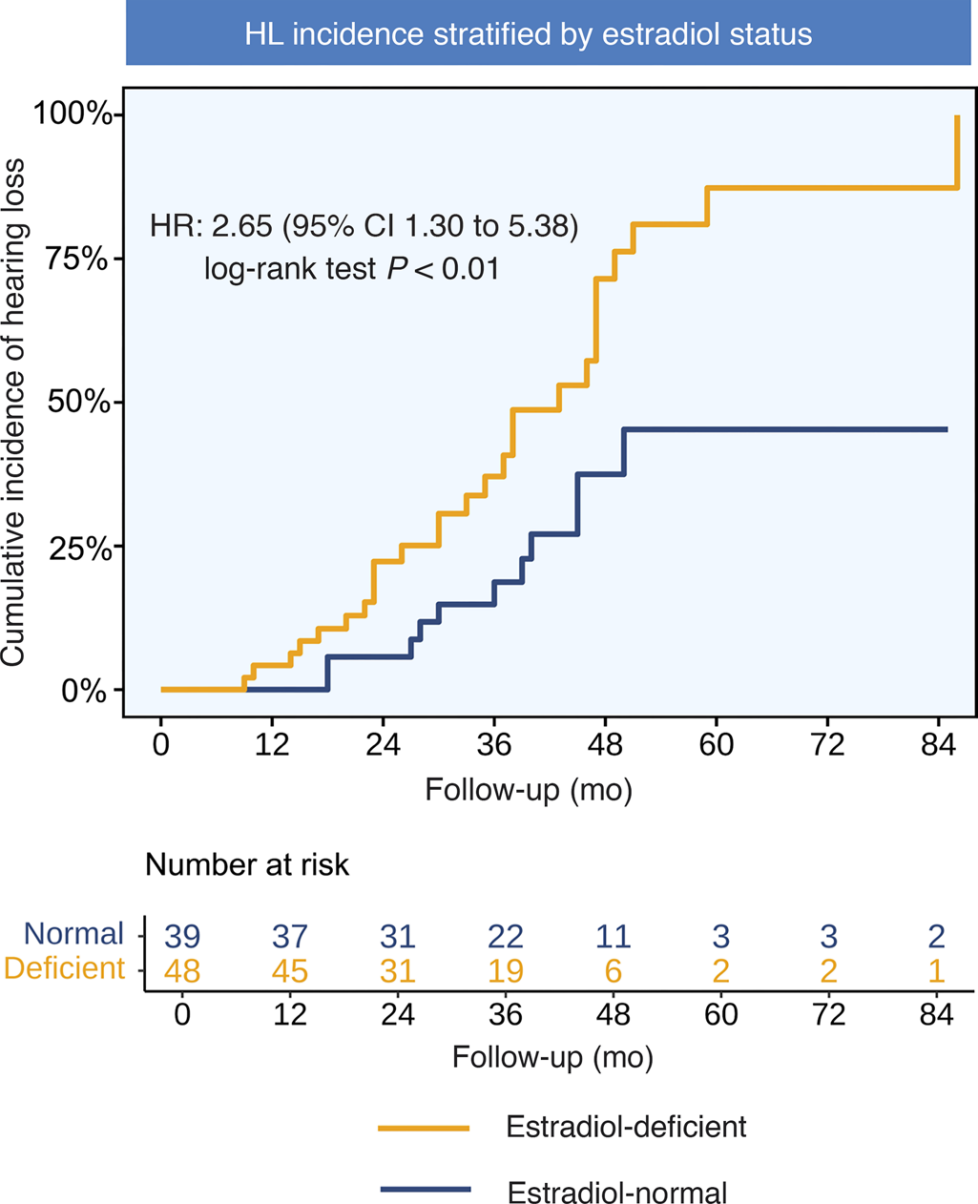
Kaplan-Meier curve of the cumulative incidence of HL. Kaplan-Meier analysis showing cumulative incidence of HL in 87 patients with TS, stratified by estrogen status. Cox regression yielded an unadjusted HR of 2.65 (95% CI 1.30–5.38); log-rank *P* < 0.01. HL, hearing loss; TS, Turner syndrome.

The analysis revealed a significant association between estrogen deficiency and incident HL over a median follow-up of 35 months (IQR 23–46 months). Among 87 patients with TS in the main cohort, 38 cases of HL were diagnosed. In estrogen-deficient individuals, the incidence of HL (27 cases, 56.3%; 20.6 events per 100 person-years [PY]) was significantly higher than that in estrogen-normal individuals (11 cases, 28.2%; 8.6 events per 100 PY). In the unadjusted analysis, estrogen deficiency conferred an increased risk of HL by 2.65-fold (95% CI 1.30–5.38; log-rank *P* = 0.005) (Figure 2). This association persisted and became more significant after stepwise adjustment of the model; after controlling for karyotype and peak growth hormone (GH) levels (Model 1), the hazard ratio (HR) remained 2.34 (95% CI 1.09–5.03; *P* = 0.031); further adjustment for IGF-1 concentration, height standard deviation score (SDS), and thyroid disorders (Model 2) yielded an HR of 2.59 (95% CI 1.12–5.98; *P* = 0.027); and in the most comprehensive model incorporating follicle-stimulating hormone (FSH) and luteinizing hormone (LH) concentrations and ovarian volume (Model 3), the risk further increased to an HR of 2.93 (95% CI 1.21–7.12; *P* = 0.019). The stable increase in the model HR value confirmed the independent effect of estrogen deficiency on the pathogenesis of HL, unaffected by growth, metabolism, and gonadal reserve factors (Table 2).

In order to further describe this association in the entire auditory spectrum, a single-frequency analysis of pure tone audiometry was performed. The results show that the effect of estrogen deficiency is more obvious at a lower frequency. The HR was highest at 500 Hz (HR 2.83, 95% CI 1.53–5.23; log-rank *P* < 0.001), followed by 1 kHz (HR 2.73, 95% CI 1.47–5.08; *P* = 0.001), 2 kHz (HR 2.11, 95% CI 1.10–4.03; *P* = 0.021), and 4 kHz (HR 2.28, 95% CI 1.14–4.56; *P* = 0.017). This pattern shows that the HR gradually decreases as the frequency increases. This suggests that estrogen plays a crucial role in maintaining low-frequency hearing in children with TS, which is vital for speech discrimination (Supplemental Figure 2).

### Reverse causality and effect modification.

**Figure 3:**
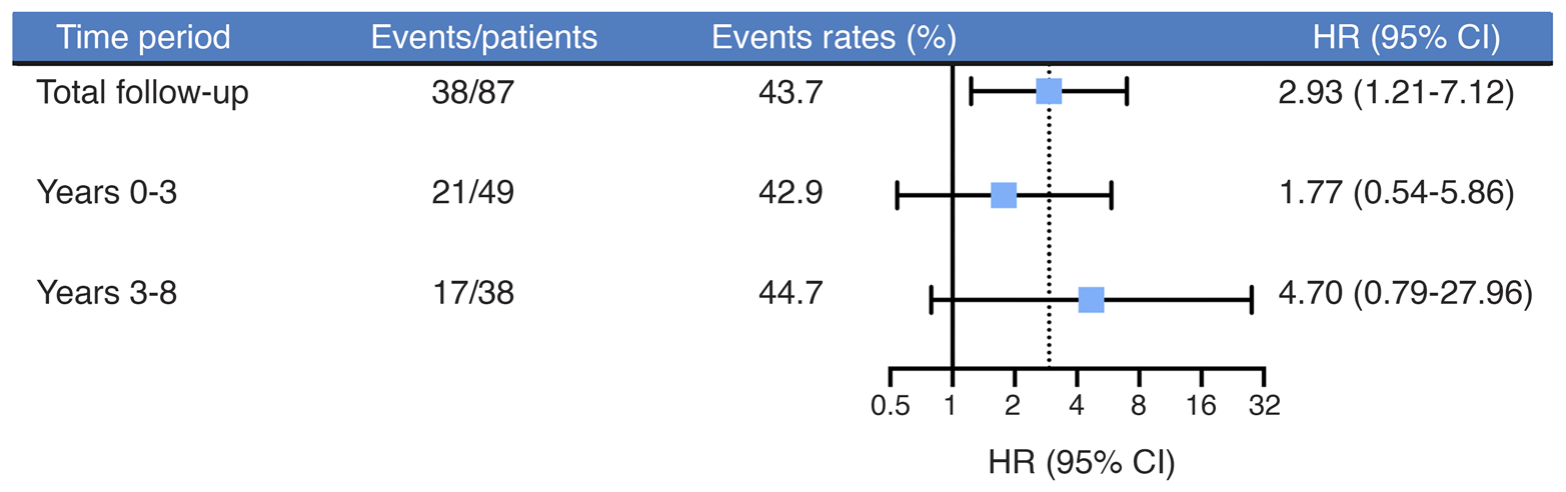
Association between estrogen deficiency and incident HL over time. HRs with 95% CIs from multivariable Cox proportional hazards models are shown for different follow-up periods. HRs were adjusted for established risk factors (Model 3 adjustment in Table 2). No corrections for multiple testing were applied. HL, hearing loss.

**Figure 4:**
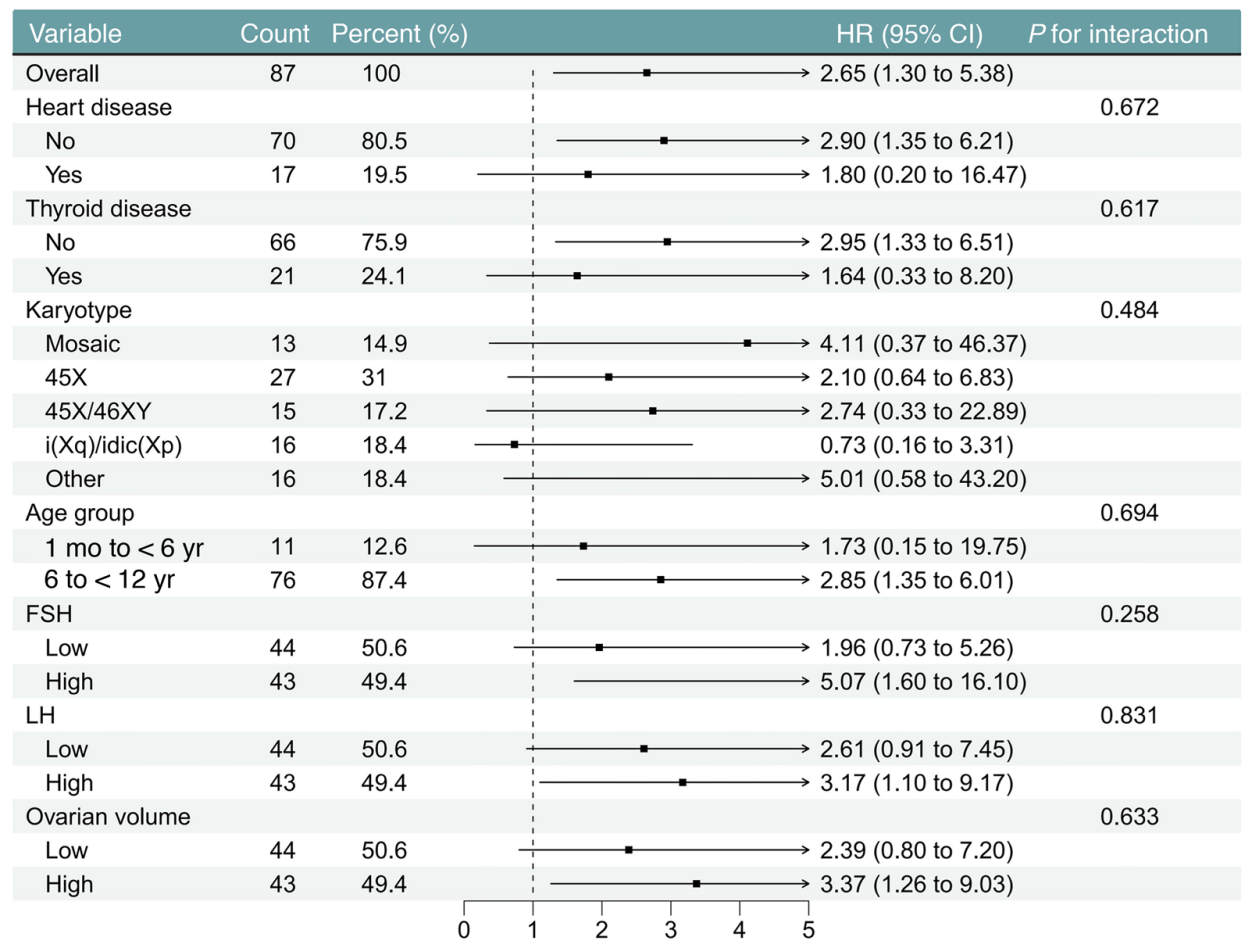
Potential effect modifiers in the association between estrogen deficiency and incident HL. This forest plot displays HRs and 95% CIs from multivariable Cox regression models across predefined subgroups. Stratification factors included baseline age, karyotype, and the presence of thyroid or cardiovascular disorders, as well as hormone levels (FSH, LH) and ovarian volume dichotomized by median values. No significant effect modification was observed across any variables (all interaction *P* values > 0.05). FSH, follicle-stimulating hormone; LH, luteinizing hormone; i(Xq)/idic(Xp), 46,X,i(Xq);46,X,idic(Xp).

To eliminate potential confounding factors and assess the robustness of the primary findings, we performed a sensitivity analysis on the main cohort. After excluding incident HL cases that occurred within 1 to 3 years after enrollment, the association between estrogen deficiency and HL remained (HR 2.53, 95% CI 1.03–6.24; Supplemental Table 2). The longitudinal analysis suggested that baseline E_2_ deficiency was associated with an increased risk of new HL. Even after 3 years of follow-up, the risk was 4.70-fold higher (95% CI 0.79–27.96) (Figure 3). Notably, this association was not significantly modified by any prespecified clinical variables, including karyotype category, age strata, markers of gonadal function, or comorbidity status (all interaction *P* > 0.05; Figure 4), confirming the robustness of our findings across different clinical subgroups.

### Combined effects of estrogen and established risk factors.

Within the main cohort, we used multivariable Cox proportional hazards models to explore the potential synergistic effect of estrogen deficiency and known comorbidities on HL risk. Specifically, we evaluated the risk of HL associated with E_2_ deficiency across each comorbidity of interest (dyslipidemia, diabetes mellitus [DM], thyroid disorders, and renal dysfunction). In the comorbidity analysis, children with TS with normal E_2_ levels and without comorbidities were used as the reference group. All models were adjusted for established risk factors.

Estrogen deficiency may increase the risk of incident HL on top of established comorbidities (Supplemental Figure 3). The HRs for the combined presence of E_2_ deficiency with dyslipidemia (HR 5.75, 95% CI 1.71–19.31), thyroid disorders (HR 2.09, 95% CI 0.72–6.09), and renal dysfunction (HR 2.00, 95% CI 0.70–5.74) exceeded those of dyslipidemia (HR 4.22, 95% CI 1.03–17.21), thyroid disorders (HR 1.05, 95% CI 0.25–4.45), and renal dysfunction (HR 0.82, 95% CI 0.23–2.91) alone. Regarding DM, the combined risk of E_2_ deficiency and DM (HR 3.89, 95% CI 1.08–14.02) appeared to be higher than that of E_2_ deficiency alone (HR 2.67, 95% CI 1.15–6.17), although it did not exceed the risk associated with DM alone. In summary, estrogen deficiency may synergistically increase the risk of HL in children with TS with dyslipidemia, renal dysfunction, and possibly thyroid disease, although these observations require confirmation in larger studies due to the limited sample sizes in the present comorbidity analyses (Supplemental Figure 3).

### Association between estrogen deficiency and HL types.

**Figure 5:**
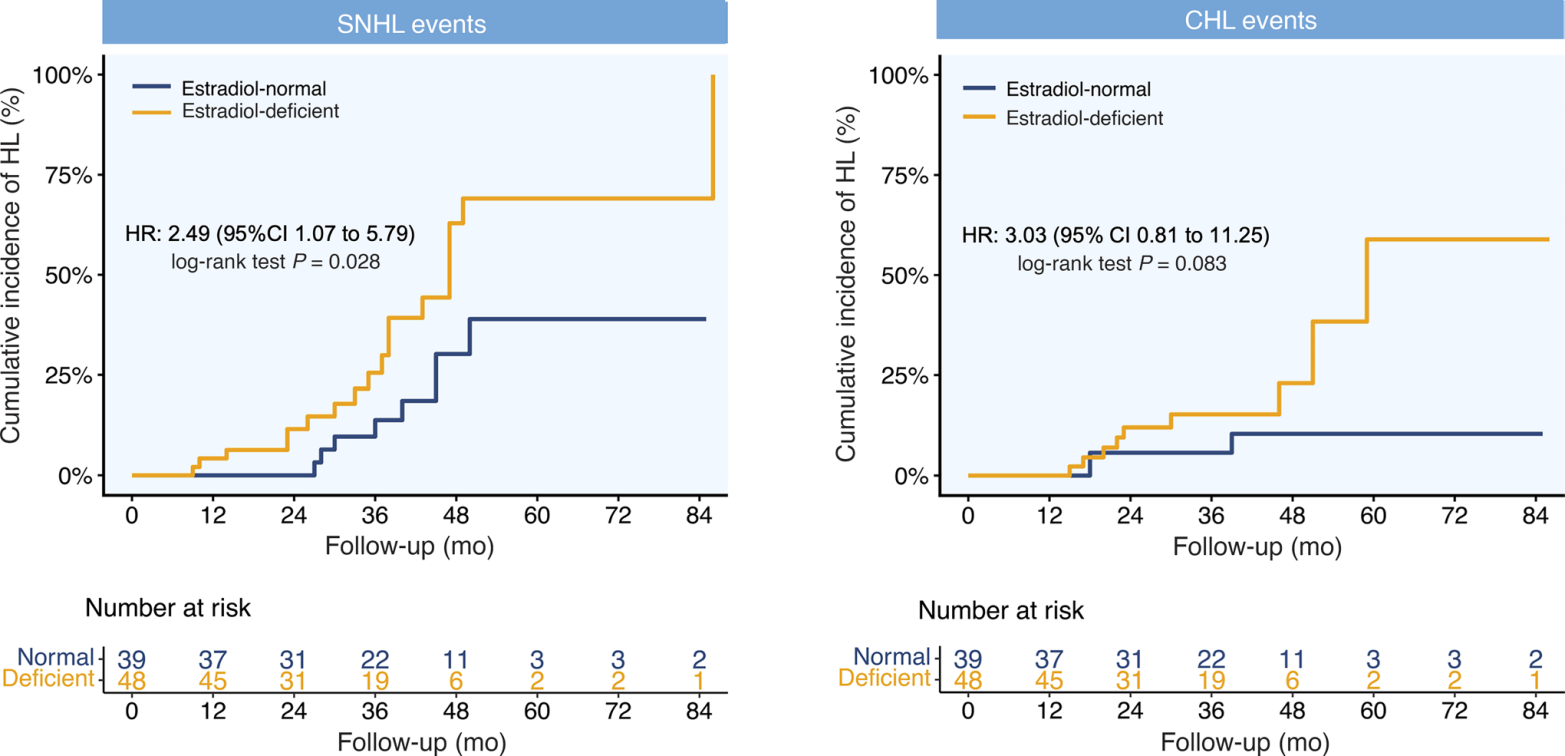
Estradiol-dependent hearing loss risk stratified by audiometric subtypes. Kaplan-Meier curves showing the cumulative incidence of sensorineural hearing loss (SNHL) and conductive hearing loss (CHL) in 87 patients with TS, stratified by estrogen status. For SNHL, Cox regression yielded an unadjusted HR of 2.49 (95% CI 1.07–5.79); log-rank *P* = 0.028. For CHL, the unadjusted HR was 3.03 (95% CI 0.81–11.25); log-rank *P* = 0.083.

Analysis of 38 incident HL cases within the TS main cohort (*n* = 87) identified SNHL as the predominant type (68.4%, *n* = 26), with CHL accounting for 31.6% (*n* = 12). By utilizing cause-specific Cox proportional hazards models to evaluate differential associations by E_2_ status, we observed that incidence rates for different types of HL were consistently higher in E_2_-deficient patients than in those with normal E_2_ levels (Figure 5).

The E_2_-deficient group exhibited a significantly higher incidence rate of SNHL (13.7 events per 100 PY) compared with the E_2_-normal group (6.2 events per 100 PY). In the unadjusted model, E_2_ deficiency was associated with a 2.49-fold increased risk of developing SNHL (HR 2.49, 95% CI 1.07–5.79). Likewise, the incidence rate of CHL was higher in the E_2_-deficient group (6.9 events per 100 PY) than in the E_2_-normal group (2.3 events per 100 PY). The unadjusted HR indicated a 3.03-fold increased risk of CHL associated with E_2_ deficiency (HR 3.03, 95% CI 0.81–11.25). Multivariable adjustment for both CHL and SNHL outcomes did not substantially alter these associations (Figure 5 and Table 3).

### Influence of abnormal DPOAEs.

**Figure 6:**
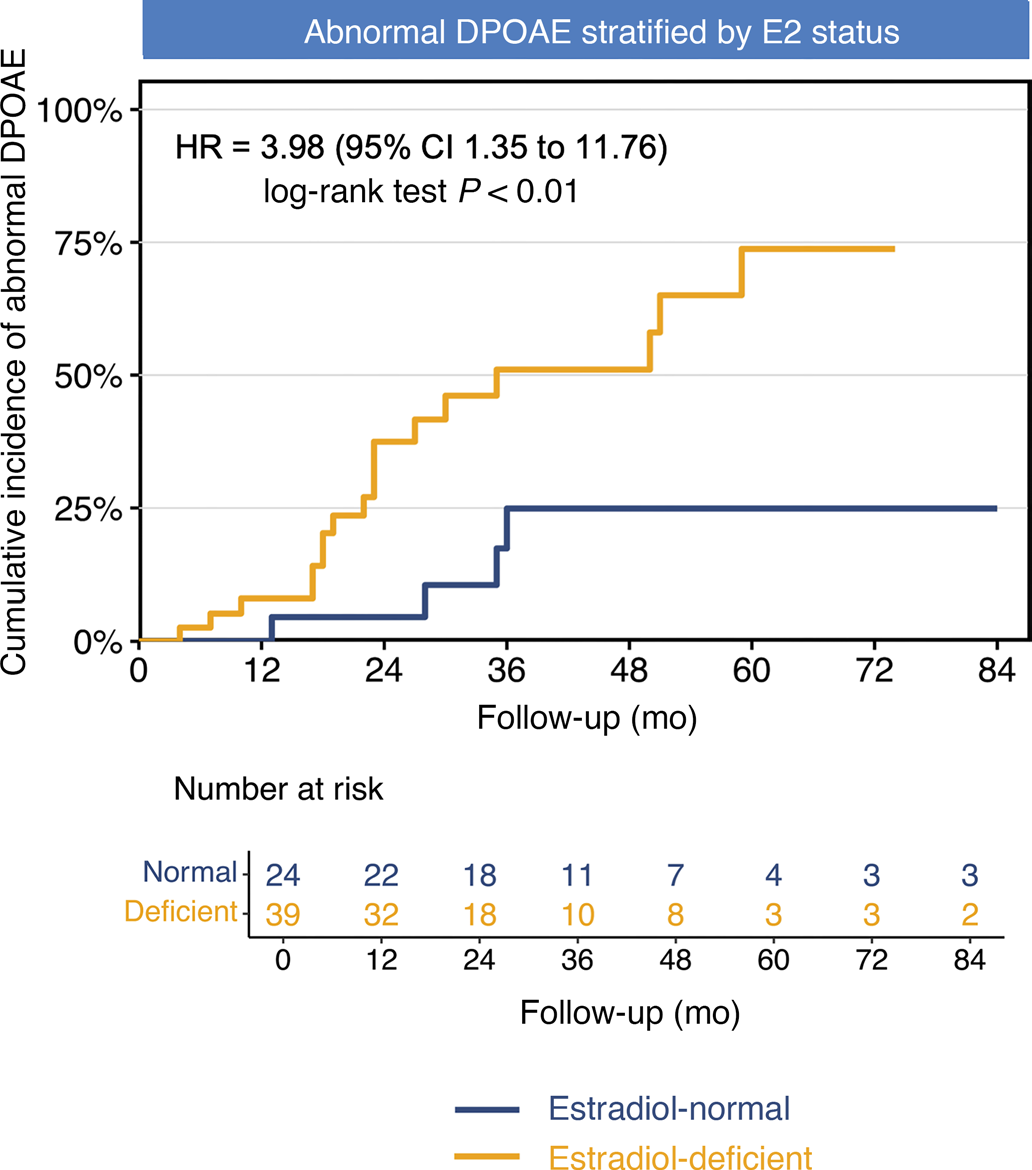
Kaplan-Meier curves of DPOAE abnormality incidence by E_2_ status. Kaplan-Meier analysis demonstrated the cumulative incidence of DPOAE abnormalities in 63 patients with TS, stratified by estrogen status. Cox regression revealed an unadjusted HR of 3.98 (95% CI 1.35–11.76; log-rank *P* < 0.01). DPOAE, distortion product otoacoustic emission.

In the main TS cohort, serial follow-up data for distortion product otoacoustic emissions (DPOAEs) were available for 63 patients, among whom 24 (38.1%) exhibited abnormal results. This observation aligns with the known high prevalence of hearing impairment in TS. Of these, 19 cases (48.7%; incidence: 19.8 per 100 PY) were observed in the E_2_-deficient group, compared with 5 cases (20.8%; incidence: 6.5 per 100 PY) in the E_2_-normal group. Notably, E_2_ deficiency independently predicted abnormal DPOAEs (HR 3.98, 95% CI 1.35–11.76; *P* = 0.007), with a greater impact than the incidence of HL (Figure 6). Moreover, further adjustments did not significantly alter the estimated HRs associated with E_2_ deficiency and abnormal DPOAEs (Supplemental Table 3).

To investigate the possibility of a frequency-specific association between DPOAE abnormalities and HL, we performed stratified Kaplan-Meier survival analysis at 9 frequencies (from 552 to 8838 Hz). In the low-frequency range, the cumulative incidence curve in the E_2_-deficient group was steeper than that in the E_2_-normal group and was significant (log-rank *P* < 0.05) at 552, 781, and 1104 Hz. Similarly, in the high-frequency range, the HR values increased, especially at 4419 Hz and 6250 Hz (both *P* < 0.05), which might reflect more obvious cochlear dysfunction at these frequencies. In contrast, the differences in the mid-frequency range seemed less significant, reaching statistical significance only at 2207 Hz (*P* = 0.022). Overall, these findings suggest a possible frequency-specific pattern, where estrogen deficiency seems to be associated with more significant DPOAE abnormalities, mainly affecting low and high frequencies, which may be related to the progressive HL pattern observed clinically (Supplemental Figure 4).

### Impact of ERT on hearing preservation in the substudy.

**Figure 7:**
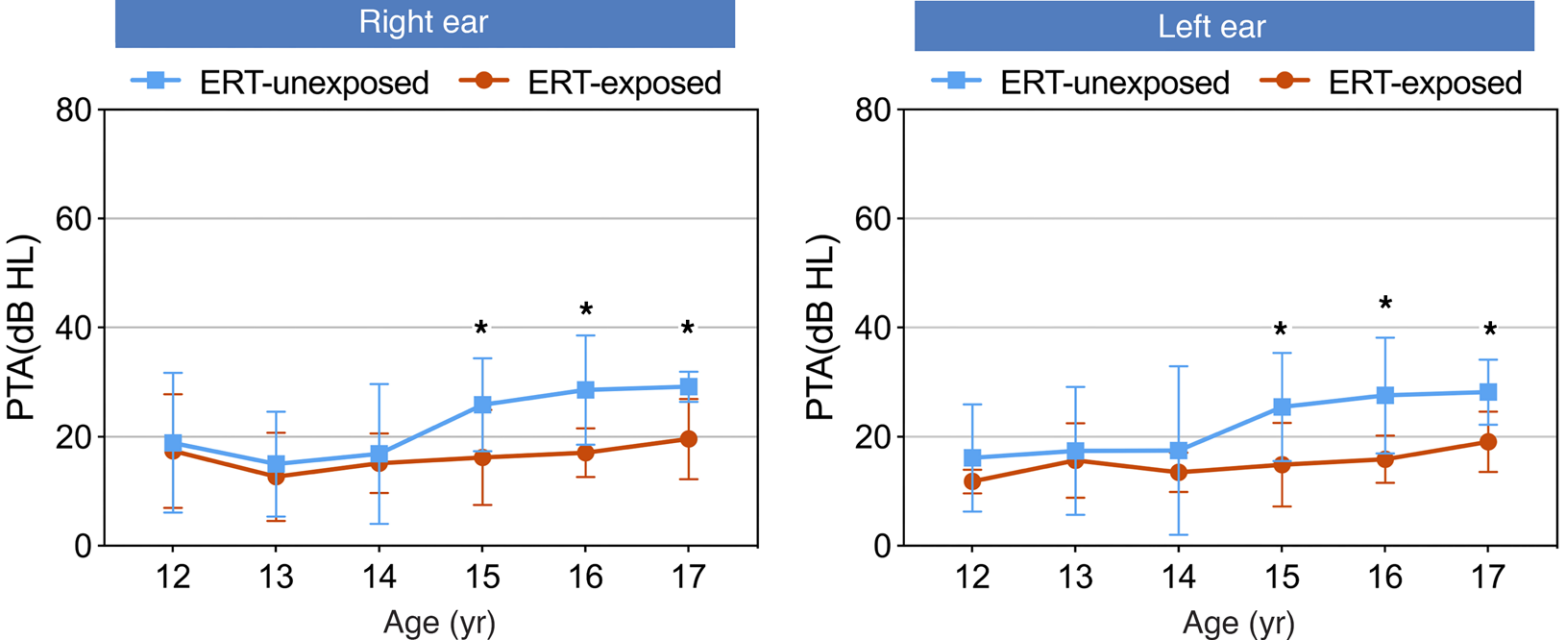
Age-banded hearing thresholds by ERT exposure status. Bilateral pure-tone average (PTA) thresholds, stratified by age and estrogen replacement therapy (ERT) exposure status, are shown. Hearing thresholds were consistently lower in ERT-exposed patients (red line) compared with unexposed counterparts (blue line) across key developmental stages. Statistical analysis using independent sample *t* tests revealed significant hearing preservation effects associated with ERT exposure at ages 15, 16, and 17 years (*P < 0.05 for all 3). Data are presented as mean ± SD; error bars indicate the dispersion of measurements around the mean. Y, years old; dB, decibel; HL, hearing level.

Given the established association between estrogen deficiency and HL in the primary study population, we sought to assess whether early ERT is associated with better hearing in the peripubertal substudy. To this end, we performed a cross-sectional analysis of hearing thresholds in 54 children with TS. For patients receiving ERT (the ERT-exposed group), the regimen consisted of oral micronized 17β-estradiol, initiated at 0.25 mg/day and titrated upwards over 2–4 years to an adult maintenance dose. Concurrent ERT was associated with major bilateral hearing preservation during key developmental stages. As illustrated in Figure 7, ERT-exposed patients consistently demonstrated lower pure-tone average (PTA) thresholds compared with their ERT-unexposed counterparts across ages 15–17 years, with statistically robust differences (all *P* < 0.05). Considerable differences were observed for both ears at age 15 (left: 14.89 vs. 25.5 dB, *P* = 0.025; right: 16.22 vs. 25.88 dB, *P* = 0.036), age 16 (left: 15.89 vs. 27.6 dB, *P* = 0.012; right: 17.11 vs. 28.6 dB, *P* = 0.011), and age 17 (left: 19.1 vs. 28.2 dB, *P* = 0.012; right: 19.6 vs. 29.2 dB, *P* = 0.016) (Figure 7).​

A total of 31 children were followed for more than 1 year (median 46 months, IQR 24–60), enabling an assessment of annual hearing decline rates. These 31 children were further divided into 20 children who began ERT between the ages of 12 and 15 years (ERT group) and 11 children who did not receive ERT during this period (Non-ERT). Baseline characteristics were generally comparable between the ERT and non-ERT subgroups. The mean age was comparable between the ERT and non-ERT groups (13.6 vs. 13.95 years). Significant differences were observed only in height SDS (ERT: –1.59 vs. Non-ERT: –2.49, *P* = 0.019) and ovarian volume (0.36 mL vs. 1.12 mL, *P* = 0.042), while all other demographic, hormonal, and physical measures showed no statistically significant differences (all *P* > 0.05) (Supplemental Table 4).

**Figure 8:**
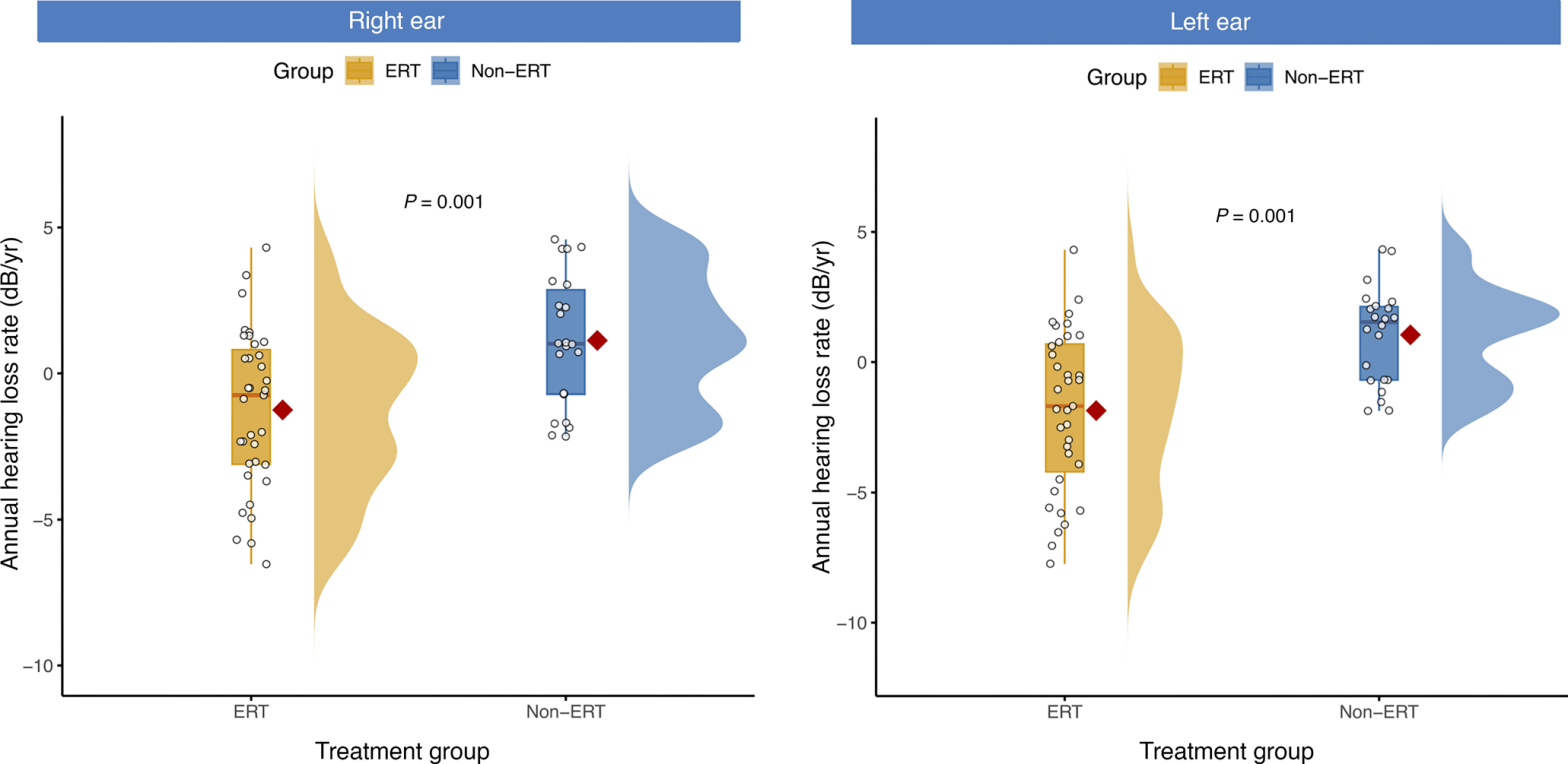
Effect of ERT on the annual hearing loss rate. This cloud plot illustrates the annual rate of hearing loss (dB/year) in patients receiving ERT versus untreated patients (Non-ERT). Each data point represents the annual rate of hearing loss for an individual ear. The plot integrates a half-violin plot (density distribution), a box-and-whisker plot (median [line], IQR [box bounds]), and raw data points. The mean for each group is indicated by a red diamond. An independent sample *t* test revealed a statistically significant difference in hearing loss progression between the 2 groups (*P* = 0.001). ERT, estrogen replacement therapy.

The longitudinal analysis of hearing trajectories demonstrated a sustained protective effect on hearing in the ERT group. The mean annual HL rate in the left ear was significantly higher in the Non-ERT group than in the ERT group (1.04 ± 1.86 vs. –1.85 ± 3.07 dB/y, *P* < 0.001). Similarly, the mean annual HL rate for the right ear was 1.13 ± 2.25 dB/y in the Non-ERT group, compared with –1.24 ± 2.72 dB/y in the ERT group (*P* = 0.001). Compared with the Non-ERT group, the annual HL rate was reduced significantly in those who initiated ERT before age 15, indicating that early intervention may alleviate long-term HL in children with TS (Figure 8).

### Sex hormone profiles and PTA association.

**Figure 9:**
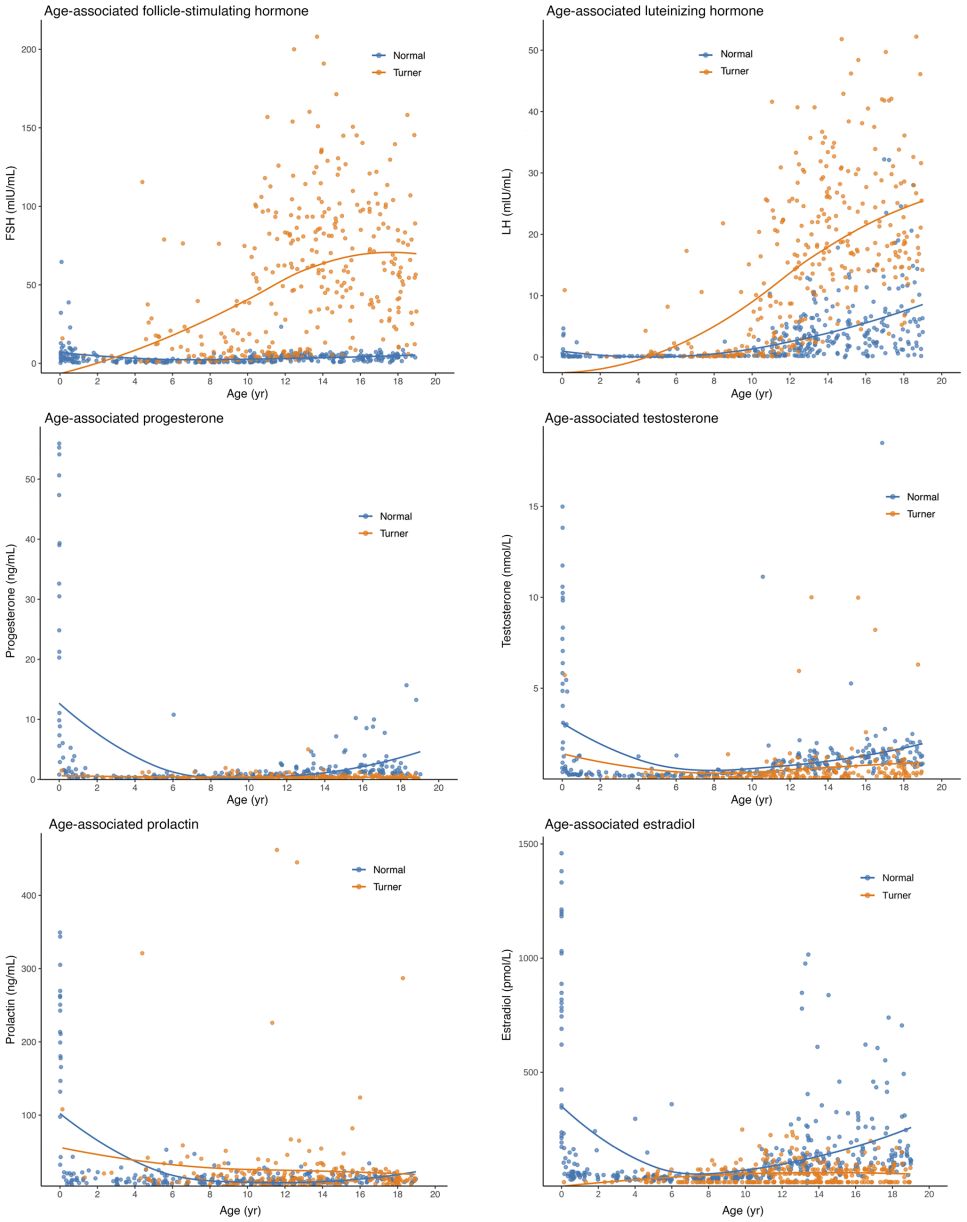
Comparative analysis of sex hormone levels: study cohort vs. age-matched healthy female participants (CALIPER Reference Intervals). This figure compares the longitudinal trajectories of 6 reproductive hormones — follicle-stimulating hormone (FSH), luteinizing hormone (LH), estradiol (E_2_), progesterone (P), prolactin (PRL), and testosterone (T) — between girls with TS and healthy controls. Individual measurements are shown as scatter points, and group trends are visualized using LOESS smoothing curves. Age-specific reference intervals for healthy girls were derived from the CALIPER database (https://caliper.research.sickkids.ca).

To elucidate the mechanism of action of ERT, we analyzed the levels of endogenous sex hormones in patients with TS in detail and assessed their association with hearing parameters. Longitudinal hormone level analysis in patients with TS revealed severely impaired gonadal function, characterized by persistently low E_2_ levels, with significantly lower E_2_ levels across all age groups (0–19 years) compared with the CALIPER healthy female control group (*P* < 0.001, Supplemental Figure 5). Notably, untreated patients with TS after age 12 typically lacked a spontaneous increase in E_2_ during puberty (*P* < 0.001 compared with CALIPER reference values). Longitudinal E_2_ level monitoring in 67 prepubertal patients with a median follow-up of 21.65 months showed limited intraindividual variability, with a median variation of 0.02 pmol/L (IQR 0.0–18.37 pmol/L). In most patients (79.2%), E_2_ levels remained stable within 0.5 standard deviations (SD) of baseline measurements (Supplemental Figures 6 and 7). Although E_2_ levels in adolescents with TS who started ERT after age 12 were higher than in untreated age-matched patients with TS (*P* = 0.008), these concentrations were still significantly lower than the physiological normal values in healthy children (*P* < 0.001; Supplemental Figure 8). This E_2_ deficiency was accompanied by systemic hypergonadotropic hypogonadism, characterized by persistently elevated FSH and LH (*P* < 0.001, Figure 9) and persistent testosterone/progesterone suppression across all ages (*P* < 0.001). Age-stratified analysis revealed different patterns of hormone-hearing associations. While circulating FSH, LH, progesterone, testosterone, and prolactin (PRL) levels showed weakly positive or no correlation with hearing thresholds across all age groups, E_2_ was the only hormone consistently negatively correlated (i.e., potentially protective), especially during puberty and beyond (11 to <19 years; *R* = –0.19 to –0.22; Supplemental Figure 9). Although these correlations did not reach statistical significance in the stratified study population, this distinct trend highlights the unique and central role of estrogen in auditory physiology relative to other reproductive hormones (Figure 9).

## Discussion

In this prospective cohort study of 87 patients with confirmed TS recruited and followed at an international tertiary referral center (with the maximum follow-up duration reaching 8 years), we demonstrated that endogenous estrogen deficiency was significantly and independently associated with an increased risk of HL development, after adjusting for established risk factors. This association remained robust for both SNHL and CHL subtypes, becoming more pronounced over time. Importantly, we further established that after the age of 12, adolescent patients at the same age exhibited significantly lower hearing thresholds if they received ERT compared with those unexposed, and longitudinal assessment over a 3-year follow-up period revealed a higher average annual hearing preservation rate in the ERT group, suggesting a potential protective effect of exogenous estrogen supplementation. Given the rarity of TS and the inherent challenges in longitudinal cohorts, the extended follow-up and comparatively substantial sample size in this study contribute to evidence suggesting that estrogen deficiency might be involved in the pathogenesis of hearing impairment in this vulnerable population. Our findings highlight the need to determine whether hormonal intervention initiated during early adolescence mitigates auditory decline, a strategy that merits further investigation.

Patients with TS typically present with progressive high-frequency SNHL, a condition similar to age-related HL but progressing more rapidly (18, 19), often accompanied by CHL. Children with TS often have eustachian tube abnormalities, such as flatter angles and narrower lumens (20). These abnormalities can interfere with middle ear ventilation, further increasing the risk of CHL in this population. DPOAEs can detect hair cell damage in the basal cochlea (high-frequency region) earlier than pure-tone audiometry (21). The main significance of DPOAE for patients with TS lies in the early detection of cochlear functional impairment. This is particularly important, as these patients may develop hearing problems from childhood (16). Notably, 38.1% of patients in the cohort had abnormal otoacoustic emissions (OAEs), reflecting impaired activity of the outer hair cells of the cochlea. Mouse models of TS have reproduced these auditory deficits, characterized by decreased sensitivity, prolonged auditory brainstem response (ABR) latency, and reduced DPOAE amplitude (22). Histopathological analysis suggests that high-frequency HL may result from the loss of hair cells in the organ of Corti at the base of the cochlea and the swelling of nerve endings beneath the hair cells (22).

The mechanisms by which estrogen deficiency leads to HL in TS involve both peripheral and central pathways. At the peripheral level, estrogen receptors (ERα and ERβ) are expressed in human organs of Corti, stria vascularis, and spiral ganglia (23). Estrogen is essential for maintaining ion homeostasis in the endolymph (24); its deficiency leads to stria vascularis atrophy and loss of outer hair cells, explaining the characteristic progressive SNHL observed in TS. At the central level, estrogen, as a neurosteroid, regulates neurotransmitter systems (such as GABA and glutamate) and promotes synaptic formation in the auditory brainstem and primary auditory cortex (25). Studies have shown that estrogen deficiency in patients with TS is associated with prolonged latency of brainstem auditory evoked potentials, indicating slower neural conduction velocity (26). Furthermore, spatial hearing tests in women with TS show mild impairment in sound source localization, which may suggest a deficit in central auditory spatial processing (16).

For patients diagnosed with TS, determining the relationship between E_2_ levels and the incidence of HL is important for the early detection of auditory dysfunction. First, estrogen deficiency is common in patients with TS, with an incidence of 55.1% in our study group, a finding consistent with the established endocrine characteristics of the syndrome (6). Second, comorbidities like diabetes and obesity may exacerbate estrogen depletion (27, 28). Beyond the high prevalence of hypoestrogenism, patients with TS face a heavy HL burden; our pediatric TS cohort demonstrated a 43.7% HL incidence rate (14.6 per 100 PY), far exceeding the 1.96% (95% CI 1.39–2.54) unilateral HL prevalence in US adolescents aged 12–19 (29). Patients with TS less than 12 years old not receiving estrogen supplementation formed the main cohort to investigate the natural association between endogenous estrogen status and incident HL. Our findings identify that estrogen deficiency (measured by E_2_ levels) is a strong and independent risk factor for HL in TS, suggesting that improving estrogen status may reduce HL risk.

Although substantial evidence supports ERT’s protective role in auditory function — as lower serum E_2_ levels correlate with reduced auditory sensitivity and postmenopausal studies confirm ERT’s efficacy in mitigating HL risk (11, 12, 30) — clinical implementation in patients with TS remains suboptimal. International guidelines recommend initiating low-dose ERT at age 12 to address hypoestrogenism (18, 31), yet in China, diagnostic delays frequently postpone treatment initiation until a median age of 15.75 years, largely due to persistent concerns about accelerated epiphyseal fusion and reduced adult height (32). In our substudy, the age at which women with TS began pubertal induction was delayed to an average of 14.75 years, mainly due to an emphasis on height optimization (average height of 126 cm at age 12), preservation of ovarian function (36.2% with FSH levels below 10 IU/L), and the presence of mosaicism (18.2%), which is consistent with conservative ERT practices in China (32).

Regarding the timing of ERT, Ross et al. demonstrated that initiating ultra-low-dose estrogen as early as age 5 could improve adult height and neurocognition (6). However, considering that early estrogen exposure could potentially accelerate epiphyseal closure and limit adult height (33), the 2016 and 2024 International Turner Syndrome Guidelines continue to recommend initiating low-dose ERT at around age 12 (31, 34). Accordingly, our substudy followed these guidelines by including patients aged 12 or more years. Whether earlier ERT (before age 12) offers superior hearing protection remains to be elucidated in future prospective studies.

This study is an extension of prior investigations on the HL of patients with TS. Population-based studies have confirmed that female patients with TS with estrogen deficiency exhibit sound localization deficits, age-dependent HL progression, and potentially elevated HL risk in those with the 45,X karyotype (16, 35). However, to date, there have been no clinical studies that can clearly elucidate the specific impact of estrogen deficiency on auditory outcomes of patients with TS. Our study fills this gap and reveals a systematic association between estrogen deficiency and the incidence of HL in patients with TS by comparing the hearing outcomes of children with TS with and without ERT during early adolescence. Future research should investigate how ERT dosing and timing modulate HL risk in TS, thereby clarifying the therapeutic potential of ERT in this population. To our knowledge, this is the first study to evaluate the causal relationship between estrogen deficiency and HL and to assess the role of ERT in hearing preservation in adolescent children with TS.

### Strengths and limitations.

While this prospective cohort study has several methodological advantages, including a long follow-up period, rigorous outcome confirmation, and limited missing data, a limitation remains; our cohort design cannot assess the effects of ERT treatment initiated before age 12. Therefore, the impact of this prepubertal treatment regimen on hearing protection in patients with TS requires further investigation.

### Conclusions.

Our longitudinal cohort study establishes that persistent estrogen deficiency in children with TS independently elevates the risk of long-term HL. Critically, early initiation of exogenous ERT during adolescence significantly attenuates this risk, underscoring the importance of timely estrogen supplementation for auditory health in patients with TS.

## Methods

### Sex as a biological variable

All patients with TS in our study were female, and sex was not considered as a biological variable.

### Study design and population

This prospective study enrolled children with TS at our center from January, 2016 to March, 2024. Inclusion criteria were the following: confirmed TS diagnosis via peripheral blood karyotyping, written informed consent obtained from legal guardians, and age under 12 years at enrollment. Exclusion criteria were as follows: (a) history of potential confounding factors for HL — exposure to ototoxic medications, history of explosive deafness or sudden exposure to intense impulse noise (e.g., from blasts, explosions), therapeutic radiation to the head and neck region, history of tumors affecting the auditory system (e.g., vestibular schwannoma), or specific infections known to cause HL (e.g., syphilis, HIV/AIDS); and (b) preexisting HL documented in prior medical evaluations. The follow-up endpoint of the main study was defined as either the onset of HL or the conclusion of the study period (March 1, 2024). Participants initially enrolled in the main study were followed longitudinally and transitioned into a predefined substudy upon reaching 12 years of age — the clinical milestone for ERT initiation. This sequential design allowed for a continuous evaluation of the same cohort, assessing the impact of endogenous estrogen status before age 12 and the influence of exogenous ERT thereafter. This substudy conducted a prospective assessment using a dual analytical framework. First, within each specific age group (12 to 17 years), a cross-sectional comparison of pure-tone hearing thresholds was performed between patients who received ERT and those who did not. Second, a longitudinal analysis was performed on participants who received at least 2 follow-ups, comparing annual changes in hearing thresholds between patients who started ERT in early adolescence (12 to 15 years) and those who did not receive treatment during this developmental stage.

### Data collection protocol

Following a standardized screening process, baseline data were collected, including chromosome karyotype, medication history, surgical history, physical examination, and laboratory findings. Karyotyping was performed in accordance with the 2016 and 2024 International Turner Syndrome Consensus Guidelines, classifying chromosomal variants [e.g., 45,X; 46,X,i(Xq); mosaicism] (31, 34). Clinical records included ERT/progestin replacement therapy, combination therapy (anabolic steroids, recombinant human GH), ototoxic medication exposure (e.g., aminoglycosides), and surgical interventions (e.g., gonadectomy). Details regarding the administration of GH and estrogen therapy are provided in the supplemental material.

### ERT protocol

ERT-exposed patients in the substudy received a standardized oral regimen. Treatment consisted of micronized 17β-estradiol (Progynova), initiated at a dose of 0.25 mg/day. The dose was titrated upwards every 6 months based on clinical assessment (breast development, uterine volume, serum E_2_ levels) over 2–4 years to reach an adult maintenance dose range of 2–4 mg/day. Safety monitoring included regular pelvic ultrasound and metabolic assessments. The complete treatment protocol is provided in the supplemental material.

### Hormone assays

Blood samples were collected from the antecubital vein in the early morning following an overnight fast. Serum concentrations of sex hormones, including E_2_, FSH, LH, testosterone, PRL, progesterone, and anti-Müllerian hormone (AMH), were quantified by chemiluminescence immunoassays (CLIA) using the Beckman Coulter UniCel DxI 800 platform (Beckman Coulter). E_2_ values are presented in pmol/L (conversion factor: 1 pg/mL = 3.671 pmol/L). The lower limit of quantification (LoQ) was 18.36 pmol/L (5 pg/mL). The follow-up schedule and monitoring indicators are detailed in the supplemental material.

### Hearing tests

#### Pure-tone audiometry.

Pure-tone audiometry and DPOAE assessments were conducted by certified audiometric technicians in a sound-attenuated booth. Air-conduction thresholds were measured using a clinical audiometer (Madsen Astera) equipped with TDH-39 supra-aural headphones (Telephonics) at octave frequencies from 0.125 to 8 kHz. Thresholds were determined binaurally according to standard clinical protocols and defined as the lowest hearing level at which reliable responses were obtained. Bone-conduction thresholds were assessed using a bone vibrator placed on the mastoid. Normal hearing was defined as a PTA at 0.5, 1, 2, and 4 kHz of less than 20 dB HL, in accordance with the World Health Organization (WHO) 2021 criteria (36). Hearing loss was defined as a PTA of 20 dB or greater HL and was further classified as CHL if an air-bone gap of 10 dB or greater HL was present, or as SNHL if the air-bone gap was less than 10 dB HL.

#### DPOAE.

DPOAEs were measured using the SmartDPOAE system (Intelligent Hearing Systems). DPOAEs were elicited using 2-tone stimulation with a fixed f_2_/f_1_ frequency ratio of 1.22 and stimulus intensities (L_1_/L_2_) of 65/55 dB SPL. The f_2_ frequencies tested were 552, 781, 1104, 1563, 2207, 3125, 4419, 6250, and 8838 Hz. The distortion product (DP) amplitude and signal-to-noise ratio (SNR) at the 2f_1_-f_2_ frequencies were recorded. A successful response at a single frequency was defined as a DP amplitude of greater than –10 dB SPL and an SNR of 6 dB or greater. An overall DPOAE test was classified as normal if at least 6 of the 9 measured frequencies met these criteria; otherwise, it was classified as abnormal (37).

All data were managed and verified through the REDCap platform (https://www.project-redcap.org/), which employs double-entry verification and automatic consistency checks. Missing values were imputed using the Multiple Imputation by Chained Equations (MICE) method. All statistical analyses and graphical representations were performed using R software (version 4.4.3; https://www.R-project.org/) and GraphPad Prism (version 10; https://www.graphpad.com/).

### Outcomes

The primary outcome for the main study cohort (TS patients aged <12 years without estrogen exposure) was incident HL, defined as the mean PTA threshold of either ear exceeding 20 dB HL for the first time during a hearing test, and this measure was obtained from a series of hearing test reports. The secondary outcome measures included abnormal DPOAE for detecting cochlear dysfunction.

The substudy comprised participants aged 12 years or older. For this cohort, we compared the bilateral PTA thresholds of the groups receiving ERT and those not receiving the therapy at matched ages (12–17 years), and evaluated the efficacy through cross-sectional landmark analysis. Longitudinal progression was quantified using the annual threshold change rate (ΔdB/year), which was calculated by dividing the consecutive threshold differences by the test interval (38).

### Clinical trial information

The number for this clinical trial is ChiCTR2300068063 (Chinese Clinical Trial Registry).

### Statistics

The age-specific E_2_ reference intervals for healthy girls were derived from the CALIPER database laboratory reference curves (available at: https://caliper.research.sickkids.ca), providing E_2_ standardized ranges stratified by sex and age. The E_2_ data of healthy females in childhood given by CALIPER Pediatric Reference Interval Database were compared with the E_2_ values of age-matched female patients with TS in the cohort (39, 40).

The cumulative incidence of HL was visualized using Kaplan-Meier curves stratified by E_2_ status. The differences between groups were evaluated by log-rank tests (41). A Cox proportional hazards model was constructed to evaluate the association between E_2_ deficiency and HL incidence in all patients with TS (*n* = 87). The model was incrementally adjusted for potential confounders: Model 1 included karyotype and peak GH levels; Model 2 added height SDS, IGF-1 concentration, and thyroid disorders; and Model 3 further adjusted for FSH/LH levels and ovarian volume. Type-specific analyses were performed for SNHL and CHL, restricted to the first documented HL event to exclude type progression. Verification using Schoenfeld residuals showed that the proportional hazards assumption was satisfied (*P* > 0.05). Tests using restricted cubic spline functions revealed no signs of nonlinearity (*P* > 0.05).

Sensitivity analysis excluded participants who developed HL within 3 years of enrollment to reduce the influence of reverse causation. Temporal consistency of the association between estrogen and HL was assessed at 3-year and 3- to 8-year intervals. The potential effect modification by enrollment age, chromosomal abnormalities, heart disease, thyroid disease, hormone levels (FSH, LH), and ovarian volume (dichotomized by median values) on the primary association was evaluated by testing interaction terms. To assess the potential synergistic effect between E_2_ deficiency and comorbidities, we investigated the combined relationship between risk factors (dyslipidemia, diabetes, thyroid disease, and renal dysfunction) and HL incidence. To address the small sample size and rare events in comorbidity combinations, we used Firth’s penalized Cox regression to ensure more robust parameter estimates.

The stability of estrogen status during follow-up was assessed by calculating the difference in E_2_ concentrations between baseline and mid-follow-up (*n* = 67). Absolute changes (follow-up value minus baseline) and standardized changes (ΔE_2_ divided by the baseline SD) were calculated. Age-stratified Spearman correlation analyses, conducted in alignment with CALIPER-defined intervals, were used to test associations between sex hormone levels and PTA thresholds. Comparison of variables between groups was performed using the unpaired *t* test or Mann-Whitney *U* test for continuous variables, and the χ^2^ test or Fisher’s exact test for categorical variables. *P* values and 95% CIs were not adjusted for multiple comparisons; therefore, the results should be interpreted with caution.

Additional statistical details are provided in the supplemental material.

### Study approval

Before sample collection, all participants signed written consent and were fully informed of sample use and test results. This study was approved by the Medical Ethics Committee of the Sun Yat-sen Memorial Hospital of Sun Yat-sen University in 2015 (SYSKY-2015-122). Participant recruitment and sample collection were conducted after obtaining ethical approval and were registered with the Chinese Clinical Trial Registry.

### Data availability

Deidentified data are available in the Supporting Data Values file. Data for study are available upon reasonable request from the corresponding authors.

## Author contributions

Zhigang Zhang and Yu Si had full access to all of the data in the study and take responsibility for the integrity of the data and the accuracy of the data analysis. Concept and design: YH, Liyang Liang, Zhigang Zhang, YS. Acquisition, analysis, or interpretation of data: All authors. Drafting of the manuscript: YH, YS. Critical review of the manuscript for important intellectual content: Liyang Liang, LZ, WL, JG, YS. Statistical analysis: ZL, Li Ling, YY, WL, Zhen Zhao. Obtained funding: Zhigang Zhang, YS. Administrative, technical, or material support: Liyang Liang, YY, LZ, ZM, YS. Supervision: Zhigang Zhang, YS.

## Conflict of interest

The authors have declared that no conflict of interest exists.

## Funding support

- National Natural Science Foundation of China grants 82173154 and 82471155.
- Fundamental Research Funds for the Central Universities, Clinical Research 5010 Program, Sun Yat-sen University (no. 2024004).

## Supplementary Material

Supplemental data

ICMJE disclosure forms

Supporting data values

## Figures and Tables

**Table 2:**
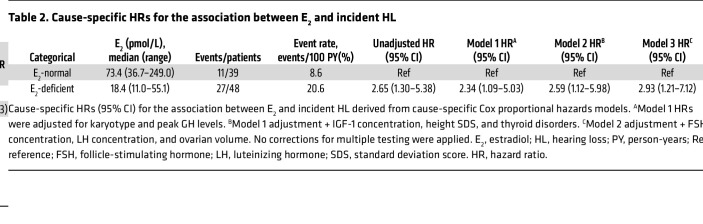
Cause-specific HRs for the association between E_2_ and incident HL

**Table 1:**
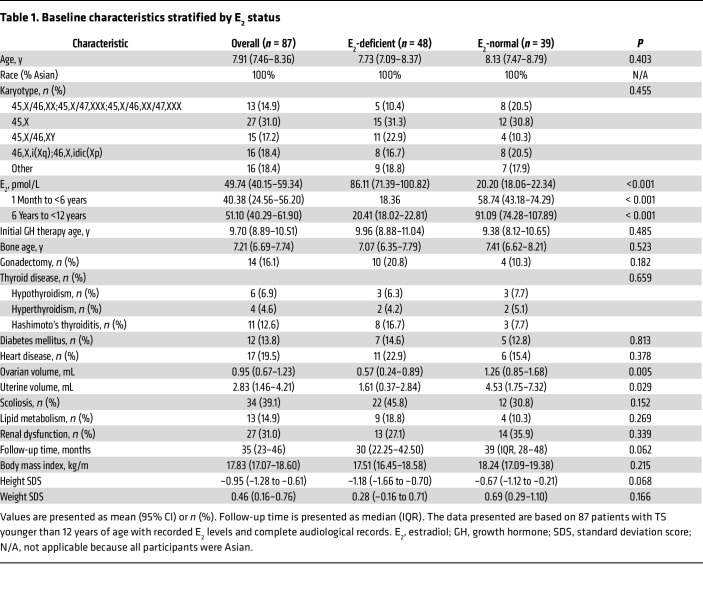
Baseline characteristics stratified by E_2_ status

**Table 3:**
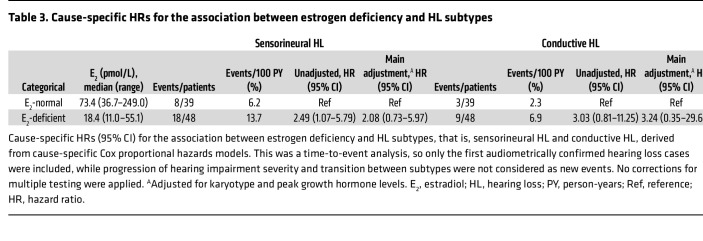
Cause-specific HRs for the association between estrogen deficiency and HL subtypes

## Version 1 03/03/2026

In-Press Preview

## Version 2 05/01/2026

Electronic publication
