# Supplementary material for: Estrogen deficiency and risk of hearing loss in pediatric Turner syndrome
Source: J Clin Invest. 2026 Mar 3;136(9):e197932. doi: 10.1172/JCI197932 (PMC13132396; doi:10.1172/JCI197932)
Supplement: Supplemental data [file jci-136-197932-s072.pdf]

Supplemental data

Title: Estrogen Deficiency and Risk of Hearing Loss in Pediatric Turner Syndrome

Table of contents (*use Ctrl + Click / ⌘ + Click to navigate*)

Protocol for Growth Hormone and Estrogen Therapy in Turner Syndrome 2

Integrated Follow-up Protocol 3

Supplemental Table 1. Baseline Laboratory values Stratified by status of Estradiol 5

Supplemental Table 2. Reverse causality assessment 6

Supplemental Table 3. Association between estrogen deficiency and abnormal DPOAE 7

Supplemental Table 4. Baseline Characteristics Stratified by status of ERT 8

Supplemental Figure 1. Distribution of Estradiol in the study population 9

Supplemental Figure 2. Kaplan-Meier Estimates of the Cumulative Incidence of Hearing Loss at Speech Frequencies. 10

Supplemental Figure 3. Combined Effects of E2 deficiency and Other Risk Factors for Incident HL . 11

Supplemental Figure 4. Cumulative Incidence of Abnormal DPOAE at Nine Test Frequencies in Turner Syndrome Patients by Estrogen Status 12

Supplemental Figure 5. Serum Estradiol Concentrations in Girls with Turner Syndrome versus Age-Matched Healthy Controls 13

Supplemental Figure 6. Distribution of the change between baseline and Midterm Serum Estradiol measurement 14

Supplemental Figure 7. Standardized change over time in Estradiol 15

Supplemental Figure 8. Age-Specific Estrogen Dynamics in TS patients 16

Supplemental Figure 9. Association Between PTA and Sex Hormones 17

Protocol for Growth Hormone and Estrogen Therapy in Turner Syndrome

This study was a single-center, prospective cohort study, and the study flow chart is shown in Fig S1.

I. Growth Hormone Therapy Protocol

Girls with karyotypically confirmed TS and growth failure (height ≤ -2.0 SDS or a decline in height velocity) received rhGH therapy.Treatment commenced at 4–6 years of age based on auxological criteria. The starting dose was 45–50 µg/kg/day, delivered via daily subcutaneous injection. Dose adjustments were implemented every 3–6 months if annual height velocity remained below the 25th percentile for TS-specific growth curves, with escalation to a maximum of 68 µg/kg/day for suboptimal responders.

Therapy was discontinued when one or more termination criteria were met: (1) radiographic bone age ≥14 years confirmed by annual hand/wrist radiography, (2) annualized height velocity <2 cm/year despite maximal dosing, or (3) attainment of satisfactory adult height (defined as height SDS > -2.0 or within the target parental height range). Safety monitoring included quarterly anthropometric assessments (height/weight plotted on TS-specific growth charts), serum IGF-1 measurements every 3 months (doses were reduced if levels exceeded +2.5 SDS to mitigate metabolic risks), and annual evaluations of fasting glucose, thyroid function, and bone age progression.

II. Estrogen Replacement Therapy Protocol

TS patients in substudy who received ERT followed this regimen.The treatment protocol utilized oral micronized 17β-estradiol (Progynova®) starting at 0.25 mg/day (equivalent to 1/10–1/8 of adult replacement dose). Dose escalation was implemented every 6 months based on clinical indicators: breast development (Tanner staging), uterine volume growth on pelvic ultrasound, and serum estradiol levels. Doses were increased by 25–50% per adjustment cycle over 2–4 years until achieving the adult maintenance range of 2–4 mg/day. Evening administration was standardized to align with physiological circadian estrogen rhythms.

Cyclic progestin add-back therapy was initiated upon meeting any of the following criteria: (1) endometrial thickness ≥5 mm on annual pelvic ultrasound, (2) occurrence of breakthrough bleeding, or (3) completion of 24 months of unopposed estrogen exposure. The progestin regimen consisted of either micronized progesterone 200 mg/day (Utrogestan®) or dydrogesterone 10 mg/day (Duphaston®) administered for 10–12 consecutive days per month. Participants received continuous daily oral estradiol (days 1–28) with overlapping progestin on days 15–26 to simulate physiological cycling and induce regular withdrawal bleeding.

Safety surveillance included baseline and annual pelvic ultrasounds quantifying uterine volume (therapeutic target: >5 mL after Year 2) and endometrial thickness (intervention threshold: ≥5 mm). Metabolic monitoring comprised liver function tests (ALT/AST) every 6 months, annual lipid profiles (LDL maintained <130 mg/dL), and quarterly blood pressure assessments.

Integrated Follow-up Protocol

Longitudinal monitoring was conducted at predetermined intervals to evaluate disease progression and therapeutic responses. Quarterly assessments included anthropometric measurements (height, weight, BMI) using calibrated stadiometers and digital scales, with standard deviation scores (SDS) calculated against Turner syndrome-specific growth charts. Biannual evaluations encompassed: (1) skeletal maturation assessment through left-hand radiography interpreted via the Greulich-Pyle atlas to guide growth hormone dose adjustment; (2) biochemical profiling comprising metabolic markers (fasting insulin, glucose, lipid panel: total cholesterol, LDL-C, HDL-C, triglycerides), hepatic/renal function (ALT, AST, BUN, creatinine), thyroid function (TSH, FT4, TPOAb, TgAb), and gonadal hormones (FSH, LH, testosterone, progesterone, prolactin quantified by chemiluminescence immunoassays on Beckman Coulter UniCel DxI 800 platform).

Annual comprehensive investigations involved: (1) cardiac Doppler echocardiography for aortic root diameter measurement, valve integrity evaluation, and ventricular function analysis; (2) pelvic ultrasound documenting uterine volume and ovarian morphology (streak gonad identification); (3) Hearing was assessed annually using a comprehensive audiological battery performed in soundproof booths, encompassing two key modalities: (1) Pure-tone audiometry (PTA) testing air conduction thresholds across 0.25–8 kHz(0.125, 0.25, 0.5, 1, 2, 4, 8 kHz) and bone-conduction thresholds at 0.5–4 kHz, utilizing a clinical audiometer (Astera, Otometrics, Denmark) to calculate mean thresholds at 0.5, 1, 2, and 4 kHz; (2) DPOAE were measured using two-tone stimulation (f_1 / f_2 = 1.22) across nine f_2 frequencies (552–8838 Hz), with a normal result defined as ≥ 6 frequencies meeting the criteria of DP > -10 dB SPL and SNR ≥ 6 dB; (3)Hearing loss classification adhered strictly to WHO 2021 criteria: conductive loss (air-bone gap ≥10 dB HL with elevated air-conduction thresholds but normal bone conduction), sensorineural loss (air-bone gap <10 dB HL with elevated thresholds in both conduction modalities)(4). Additionally, HbA1c and 25-hydroxyvitamin D levels were assessed annually to monitor metabolic bone health and diabetes risk.

	Items	Baseline	3M±2w	6M±2w	9M±2w	12M±2w	...(Similar annual)
Enrollment	Eligibility screening	√					
	Informed consent	√					
	Karyotype	√					
	Ototoxic drug exposure	√					
	Medical history collection	√					
Assessments	Height/Weight/BMI SDS	√	√	√	√	√	Quarterly
	IGF1/IGF-BP3	√	√	√	√	√	Quarterly
	Bone Age	√		√		√	Half-yearly
	Biochemical Panel	√		√		√	Half-yearly
	Gonadal Hormones	√		√		√	Half-yearly
	Cardiac Ultrasound	√				√	Annually
	Pelvic Ultrasound	√				√	Annually
	Audiological Battery	√				√	Annually

The schedule of the trial

Supplemental Table 1. Baseline Laboratory values Stratified by status of Estradiol				
Characteristic	**Overall (n=87)**	**E2 deficiency(n=48)**	**E2 normal(n=39)**	P
INS-Insulin, mu/L	10.22 (8.53-11.91)	8.70 (7.16-10.24)	12.14 (8.84-15.43)	0.099
Blood Glucose, mmol/L	4.60 (4.45-4.75)	4.52 (4.29-4.75)	4.71 (4.50-4.91)	0.300
Total cholesterol, mmol/L	4.88 (4.64-5.11)	4.95 (4.66-5.24)	4.77 (4.34-5.20)	0.462
LDL-cholesterol, mmol/L	2.79 (2.61-2.96)	2.79 (2.58-3.00)	2.78 (2.46-3.10)	0.960
HDL-cholesterol, mmol/L	1.57 (1.48-1.65)	1.63 (1.51-1.75)	1.47 (1.36-1.58)	0.073
Triglycerides, mmol/L	1.11 (0.90-1.31)	1.00 (0.81-1.18)	1.25 (0.83-1.68)	0.280
AST, U/L	30.43 (28.31-32.55)	31.19 (28.82-33.56)	29.32 (25.27-33.38)	0.092
ALT, U/L	23.52 (19.53-27.51)	23.01 (18.46-27.57)	24.26 (16.70-31.81)	0.557
GGT, U/L	28.45 (16.83-40.08)	28.27 (12.43-44.10)	28.88 (13.68-44.07)	0.518
Uric Acid, μmol/L	349.67 (328.33-371.01)	332.57 (305.15-359.99)	370.77 (336.98-404.56)	0.075
Creatinine, μmol/L	48.66 (46.16-51.16)	47.58 (44.16-51.00)	50.19 (46.39-53.98)	0.206
ESR, mm/h	10.38 (8.08-12.68)	10.75 (7.67-13.84)	9.50 (5.84-13.16)	0.619
FT3, pmol/L	5.82 (5.35-6.29)	6.02 (5.30-6.74)	5.57 (4.98-6.16)	0.347
FT4, pmol/L	34.09 (25.02-43.17)	34.03 (21.91-46.14)	34.18 (19.79-48.56)	0.588
TSH, mU/L	3.52 (2.87-4.17)	3.46 (2.60-4.33)	3.59 (2.56-4.61)	0.912
TPOAb, U/mL	289.55 (120.30-458.79)	280.03 (102.45-457.61)	303.14 (-42.62-648.91)	0.780
TgAb, IU/mL	119.79 (52.71-186.87)	86.03 (9.52-162.55)	166.40 (40.98-291.82)	0.437
FSH, mIU/mL	47.18 (37.10-57.26)	48.83 (35.17-62.49)	45.16 (29.56-60.75)	0.639
LH, mIU/mL	10.85 (8.42-13.29)	11.97 (8.59-15.35)	9.44 (5.83-13.05)	0.470
Testosterone, nmol/L	0.42 (0.21-0.63)	0.34 (0.03-0.66)	0.55 (0.30-0.79)	0.001
Progesterone, ng/mL	0.45 (0.35-0.56)	0.40 (0.29-0.52)	0.53 (0.32-0.73)	0.311
Prolactin, ng/mL	31.62 (14.96-48.28)	34.16 (10.93-57.39)	27.95 (3.08-52.82)	0.004
AMH, ng/ml	0.69 (0.17-1.22)	0.27 (0.01-0.53)	1.40 (0.06-2.73)	0.107
GH peak, ng/ml	7.56 (6.18-8.95)	7.83 (6.16-9.50)	7.07 (4.31-9.83)	0.346
IGF-1, ng/ml	279.99 (247.11-312.86)	300.66 (251.95-349.38)	253.40 (210.79-296.02)	0.157
IGFBP3, ng/ml	5.54 (5.23-5.85)	5.53 (5.09-5.97)	5.55 (5.09-6.02)	0.948
IGF-1 / IGFBP-3	48.89 (43.76-54.01)	51.71 (44.16-59.27)	44.91 (38.36-51.46)	0.223

Values are presented as Mean (95% confidence interval), or n (%). E2 =Estradiol; GH peak = Growth Hormone Peak; INS = Insulin; LDL = Low-Density Lipoprotein; HDL = High-Density Lipoprotein; AST = Aspartate Aminotransferase; ALT = Alanine Aminotransferase; GGT = Gamma-Glutamyl Transferase; ESR = Erythrocyte Sedimentation Rate; FT3 = Free Triiodothyronine; FT4 = Free Thyroxine; TSH = Thyroid-Stimulating Hormone; TPOAb = Thyroid Peroxidase Antibody; TgAb = Thyroglobulin Antibody; FSH = Follicle-Stimulating Hormone; LH = Luteinizing Hormone; AMH = Anti-Müllerian Hormone; IGF-1 = Insulin-like Growth Factor 1; IGFBP3 = Insulin-like Growth Factor Binding Protein 3.

Supplemental Table 2. Reverse causality assessment

	Total population	Exclude events <1 year	Exclude events <2 years	Exclude events <3 years
Total Events/Patients	38/87	36/85	26/75	18/67
E2 normal HR (95% CI)	Ref	Ref	Ref	Ref
E2 deficient Unadjusted HR (95% CI)	2.65 (1.30-5.38)	2.48 (1.18-5.22)	2.26 (0.95-5.35)	2.46 (0.83-7.24)
E2 deficient Adjusted HR (95% CI)	2.93 (1.21-7.12)	2.53 (1.03-6.24)	1.58 (0.47-5.23)	1.96 (0.23-16.66)

Sensitivity analyses were conducted for estrogen deficiency-associated hearing loss by excluding events occurring within 1, 2, or 3 years after enrollment. Hazard ratios were adjusted for karyotype, peak growth hormone levels, IGF-1 concentration, height SDS, thyroid diseases, FSH concentration, LH concentration, and Ovarian volume. No corrections for multiple testing were applied. E2, Estradiol; Ref, reference; HR, Hazard Ratio; CI, Confidence Interval.

Supplemental Table 3. Association between estrogen deficiency and abnormal DPOAE

Categorical	E2 (pmol/L), Median (Range)	Events/Patients	Event Rate, Events/100 PY(%)	Unadjusted HR(95% CI)	Model 1 HR[a] (95% CI)	Model 2 HR[b] (95% CI)	Model 3 HR[c] (95% CI)
E2-Normal	73.4 (36.7-249.0)	5/24	6.5	Ref	Ref	Ref	Ref
E2-Deficient	18.4 (11.0-47.6)	19/39	19.8	3.98 (1.35-11.76)	3.78 (1.09-13.15)	3.44 (0.93-12.66)	3.23 (0.82-12.71)

Cause-specific HRs (95% CI) for the association between Estradiol and incident HL derived from cause-specific Cox proportional hazards models. [a] Model 1 Hazard ratios were adjusted for karyotype, peak growth hormone levels. [b] Model 1 adjustment + IGF-1 concentration, height SDS, and thyroid disorders. [c] Model 2 adjustment + FSH concentration, LH concentration, and Ovarian volume. No corrections for multiple testing were applied. E2, Estradiol; DPOAE, Distortion Product Otoacoustic Emissions; PY, person-years; Ref, reference; HR, Hazard Ratio; CI, Confidence Interval.

Supplemental Table 4. Baseline Characteristics Stratified by status of ERT				
Characteristic	**Overall (n=31)**	**ERT (n=20)**	**Non-ERT (n=11)**	P
Age	13.73 (13.27-14.19)	13.60 (13.22-13.98)	13.95 (12.72-15.18)	0.302
Karyotype, n(%)				0.431
45,X/46,XX;45,X/47,XXX; 45,X/46,XX/47,XXX	5 (16.1%)	3 (15.0%)	2 (18.2%)	
45,X	10 (32.3%)	8 (40.0%)	2 (18.2%)	
45,X/46,XY	5 (16.1%)	3 (15.0%)	2 (18.2%)	
46,X,i(Xq);46,X,idic(Xp)	5 (16.1%)	4 (20.0%)	1 (9.1%)	
Other	6 (19.4%)	2 (10.0%)	4 (36.4%)	
E2, pmol/L	55.18 (38.90-71.47)	48.17 (33.34-63.01)	66.65 (27.56-105.74)	0.694
FSH, mIU/mL	72.09 (53.89-90.30)	74.68 (59.66-89.70)	67.86 (21.16-114.56)	0.763
LH, mIU/mL	18.05 (14.00-22.11)	19.31 (15.41-23.21)	16.00 (6.34-25.66)	0.495
Testosterone, nmol/L	0.39 (0.24-0.54)	0.37 (0.18-0.56)	0.41 (0.14-0.69)	0.777
Progesterone, ng/mL	0.47 (0.30-0.64)	0.55 (0.24-0.85)	0.39 (0.19-0.58)	0.334
Prolactin, ng/mL	19.25 (12.90-25.59)	18.66 (9.46-27.87)	19.89 (9.22-30.57)	0.673
FSH <10 IU/mL, n(%)	4 (12.9%)	0	4 (36.4%)	0.014
Gonadectomy, n(%)	4 (12.9%)	3 (15%)	1 (9.1%)	1.000
Thyroid disease, n(%)	8 (25.8%)	7 (35.0%)	1 (9.1%)	0.203
Heart disease, n(%)	8 (25.8%)	3 (15%)	5 (45.5%)	0.095
Uterine volume,ml	2.23 (0.49-3.96)	1.08 (0.24-1.93)	4.51 (-1.04-10.07)	0.061
Ovarian volume,ml	0.70 (0.35-1.05)	0.36 (0.20-0.52)	1.12 (0.40-1.84)	0.042
Scoliosis, n(%)	17 (54.8%)	9 (45%)	8 (72.7%)	0.258
Kidney function, n(%)	10 (32.3%)	7 (35.0%)	3 (27.3%)	0.675
Follow-up time (months)	46.08 (IQR, 23.80 - 60.00)	54.90 (IQR, 26.95 - 64.63)	34.92 (IQR, 23.41 - 50.07)	0.148
Body mass index, kg/m	19.05 (17.91-20.19)	19.18 (17.87-20.49)	18.82 (16.30-21.33)	0.763
Height SDS	-1.91 (-2.23 to -1.59)	-1.59 (-1.86 to -1.32)	-2.49 (-3.19 to -1.79)	0.019
Weight SDS	-0.86 (-1.31 to -0.40)	-0.72 (-1.14 to -0.29)	-1.11 (-2.26 to 0.03)	0.398

Values are presented as Mean (95% confidence interval), or n (%). Follow-up time is presented as median (IQR). E2 =Estradiol; SDS = Standard Deviation Score; ERT = estrogen replacement treatment. IQR = interquartile range.

Supplemental Figure 1. Distribution of Estradiol in the study population

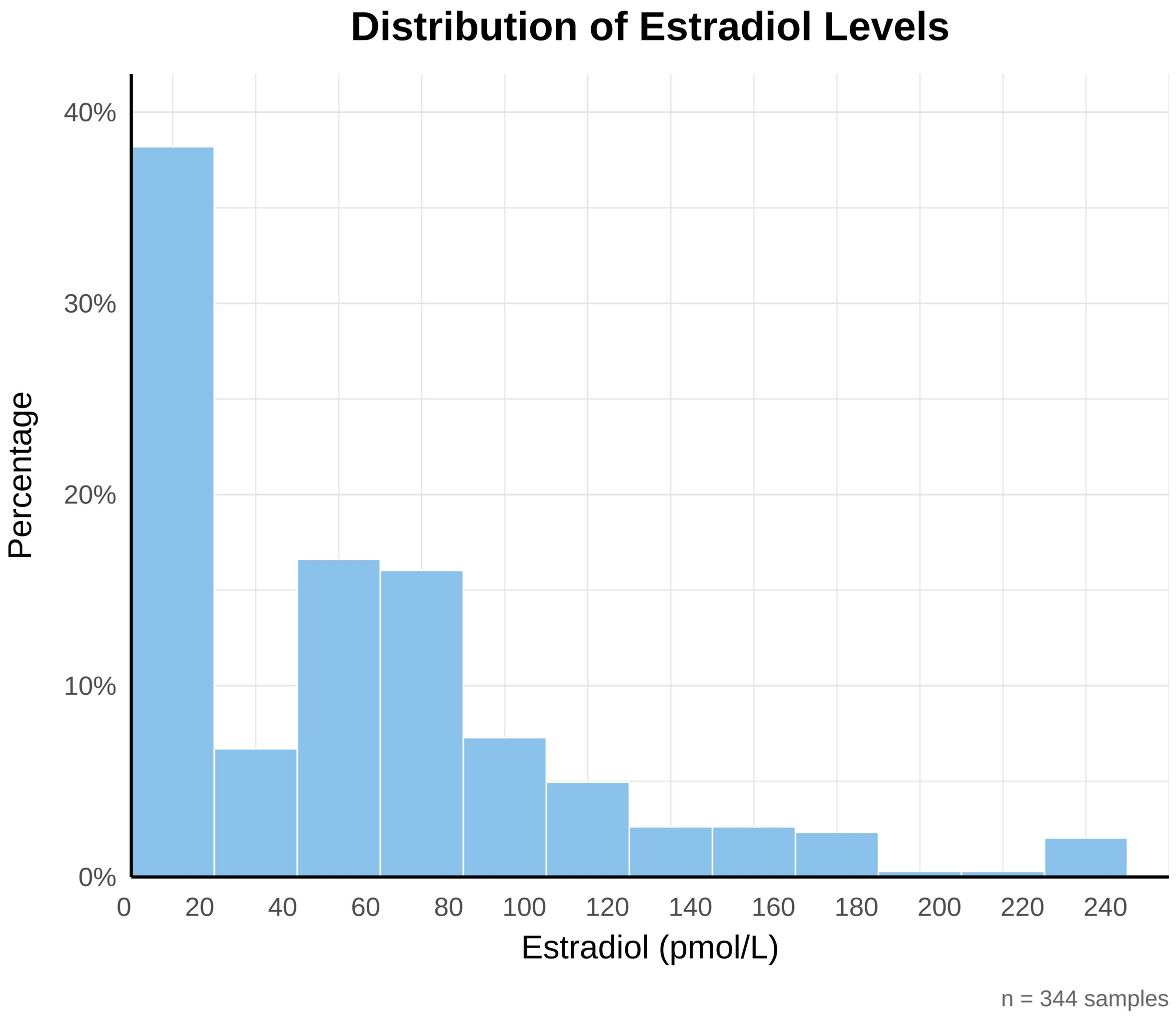

Serial measurements of estradiol levels were obtained throughout the follow-up period for all 87 Turner syndrome patients. The composite distribution encompasses all estradiol assay results from enrollment (baseline) to study termination (March 2024).

Supplemental Figure 2. Kaplan-Meier Estimates of the Cumulative Incidence of Hearing Loss at Speech Frequencies.

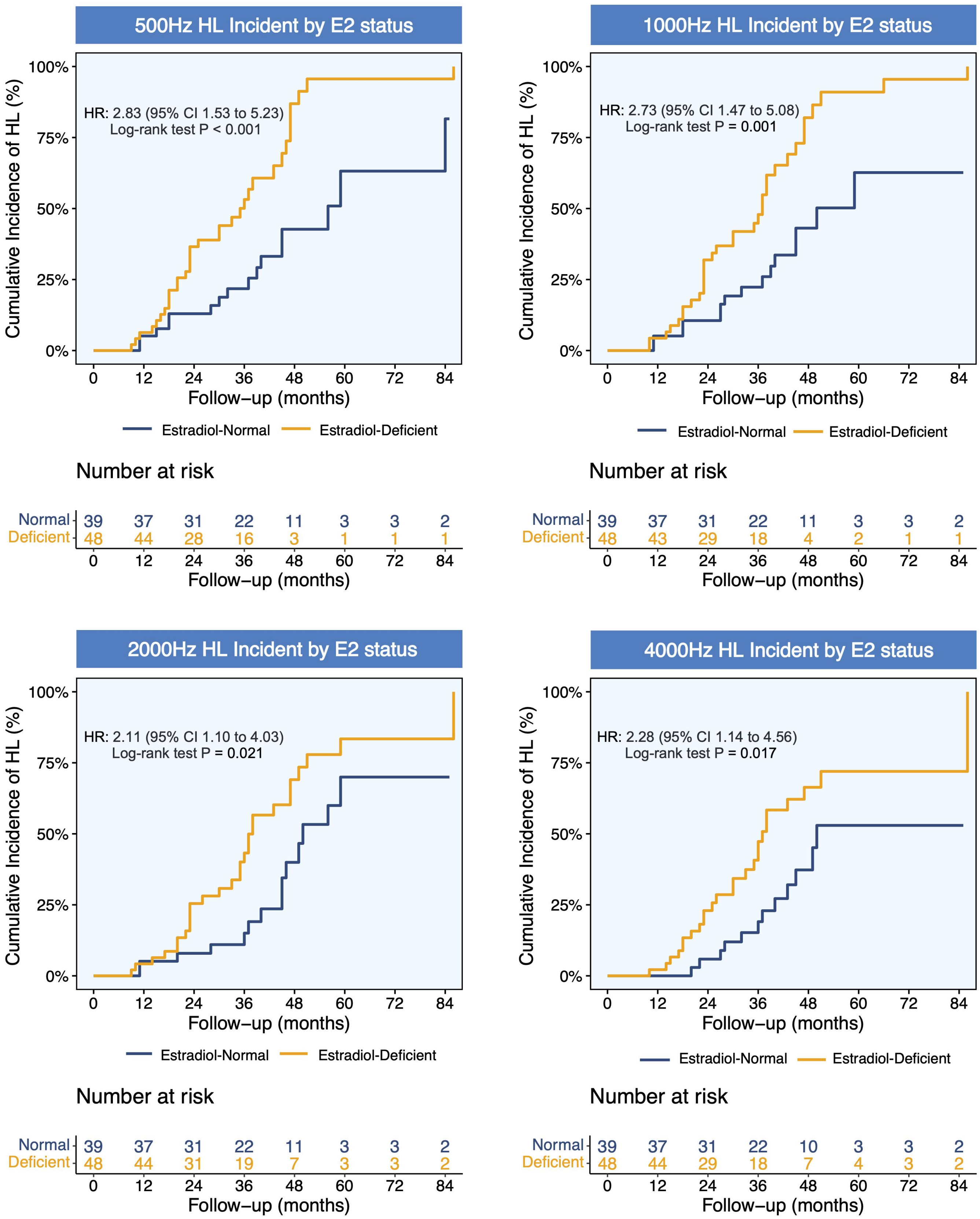

Kaplan-Meier analysis showing the cumulative incidence of hearing loss at 500, 1000, 2000, and 4000 Hz in 87 Turner syndrome patients, stratified by estrogen status. Cox regression derived hazard ratios (HRs) for estrogen deficiency are presented for each frequency. The log-rank test was used to assess the statistical significance of the differences between the groups. HL, hearing loss.

Supplemental Figure 3. Combined Effects of E2 deficiency and Other Risk Factors for Incident HL

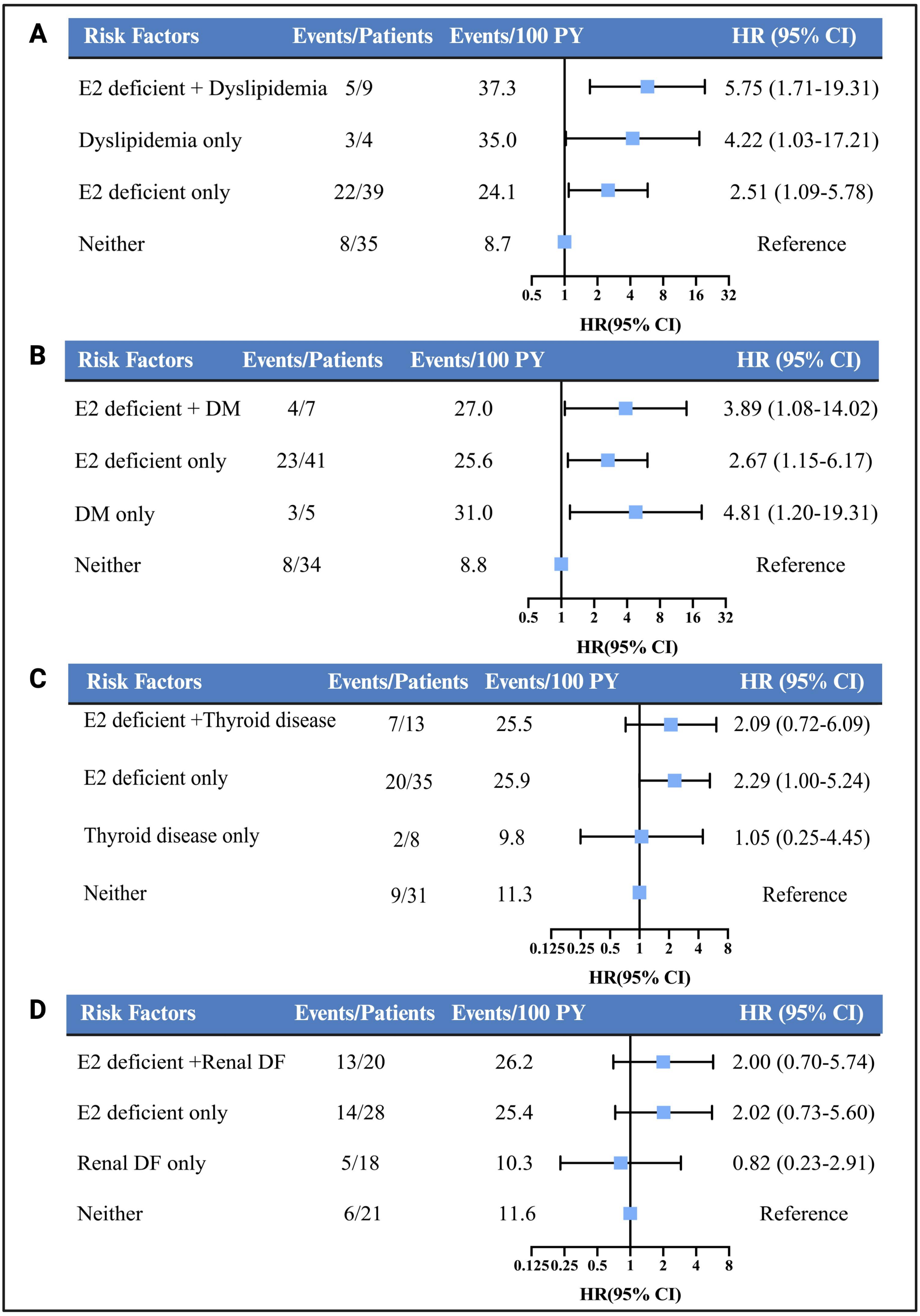

Combined effects of estradiol deficiency and dyslipidemia (A), diabetes mellitus (DM) (B) , thyroid disease (C), and Renal dysfunction (DF)(D) on the risk of incident hearing loss (HL).Hazard ratios (HRs) were derived from multivariable Cox proportional hazards regression models, adjusted for karyotype and growth hormone levels. To address potential bias due to small sample sizes or rare events, Firth's penalized maximum likelihood method was applied to correct parameter estimation bias and ensure more reliable confidence intervals. The proportional hazards assumption for each Cox model was verified using Schoenfeld residual tests, and no significant violations were detected (Global test $p > 0.05$). No corrections for multiple testing were applied. HL, hearing loss; HR, hazard ratio; CI, confidence interval; PY, person-years.

Supplemental Figure 4. Cumulative Incidence of Abnormal DPOAE at Nine Test Frequencies in Turner Syndrome Patients by Estrogen Status

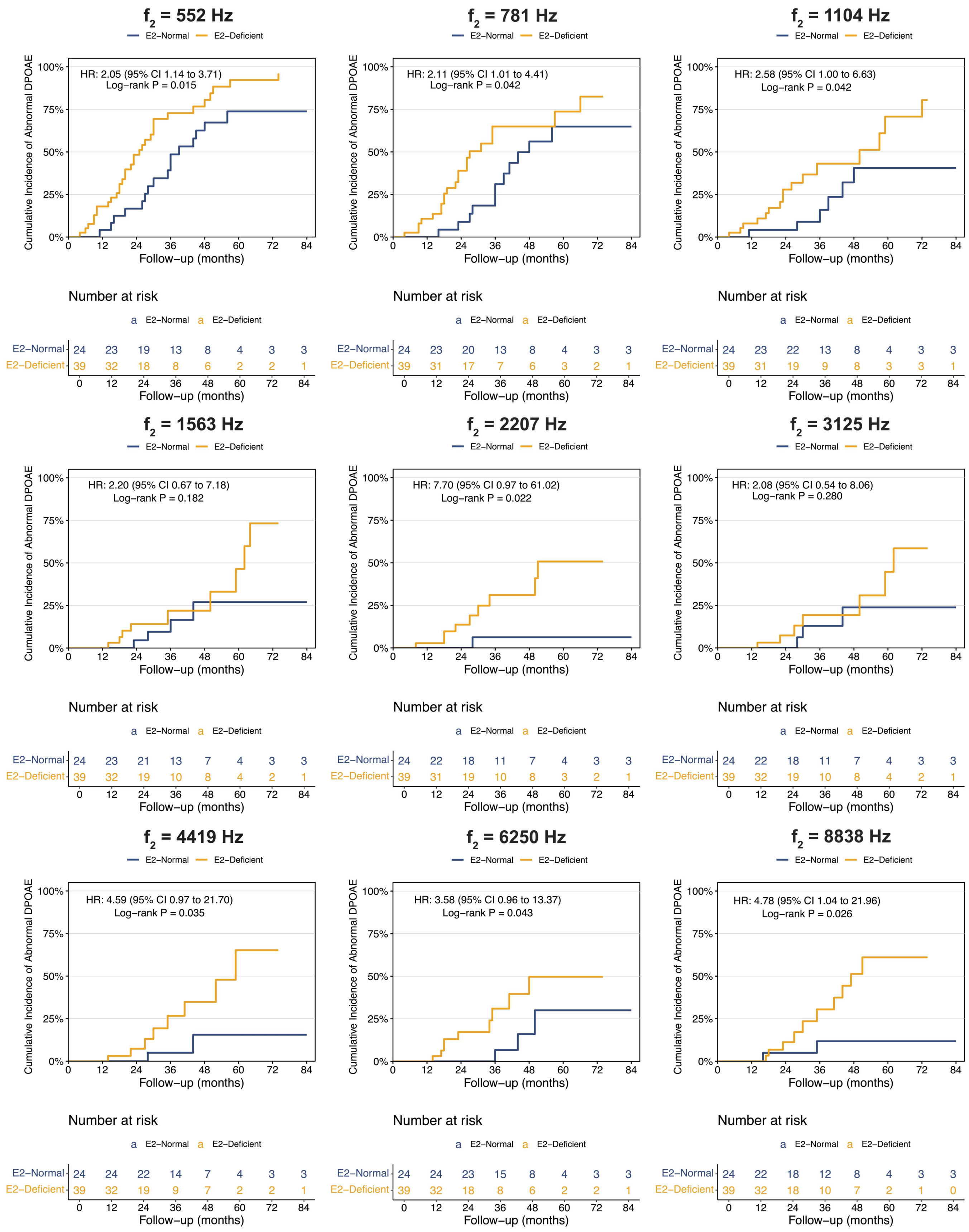

Kaplan-Meier curves show the cumulative incidence of abnormal DPOAE across nine standard frequencies (f2: 552–8838 Hz) in 87 Turner syndrome patients, stratified by estrogen status. A result was considered normal if the distortion product (DP) amplitude was > -10 dB SPL with a simultaneous signal-to-noise ratio (SNR) ≥ 6 dB. Conversely, failure to meet either of these criteria was classified as an abnormal result at that frequency.Unadjusted hazard ratios (HR) with 95% confidence intervals (CI) from Cox proportional hazards models are reported for each frequency. DPOAE, distortion product otoacoustic emissions; SNR, signal-to-noise ratio; SPL, sound pressure level.

Supplemental Figure 5. Serum Estradiol Concentrations in Girls with Turner Syndrome versus Age-Matched Healthy Controls

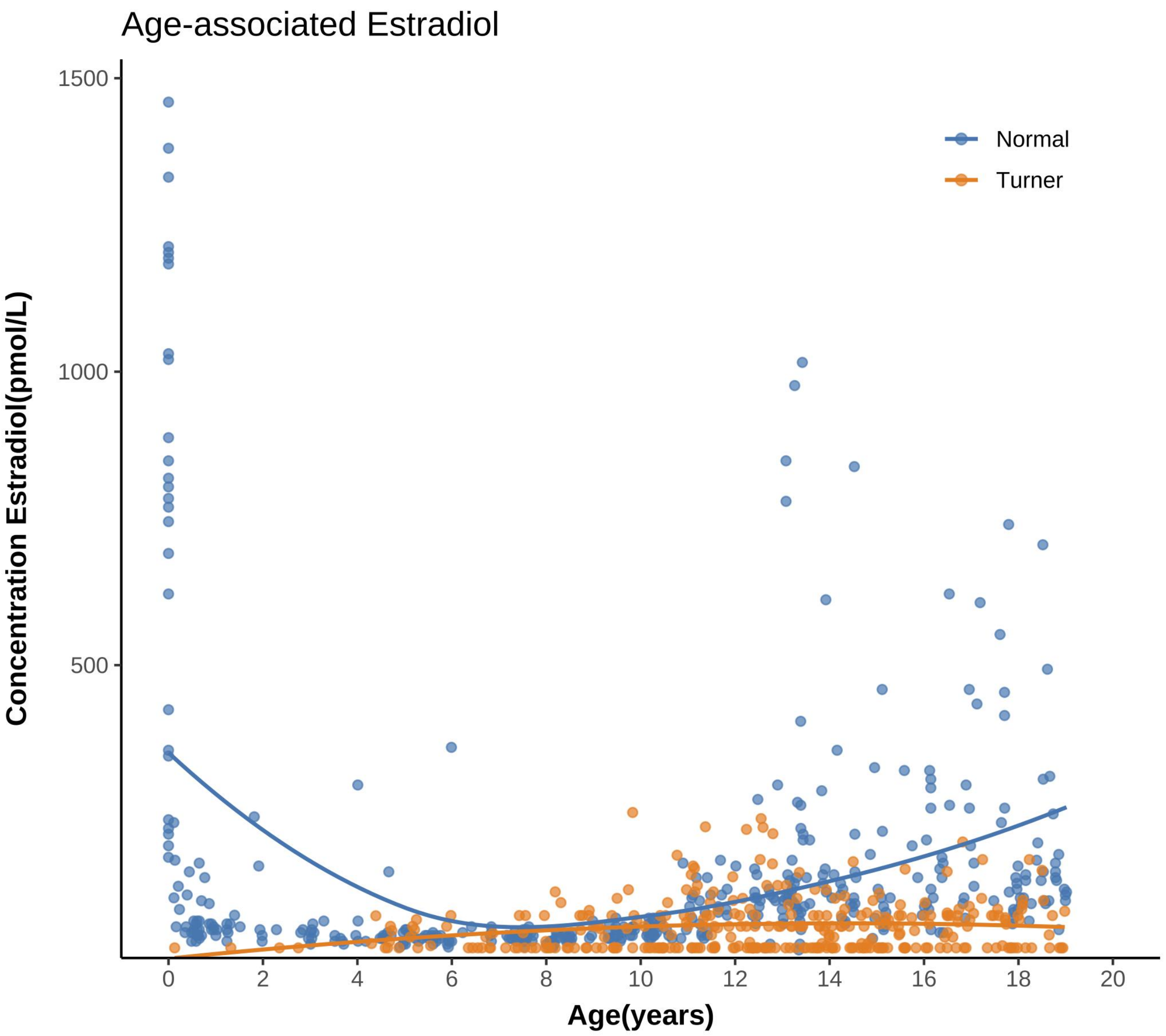

Serum estradiol levels in Turner syndrome (TS) patients versus age-matched healthy controls (longitudinal cohort analysis). Control data sourced from the CALIPER Pediatric Reference Interval Database.

Supplemental Figure 6. Distribution of the change between baseline and Midterm Serum Estradiol measurement

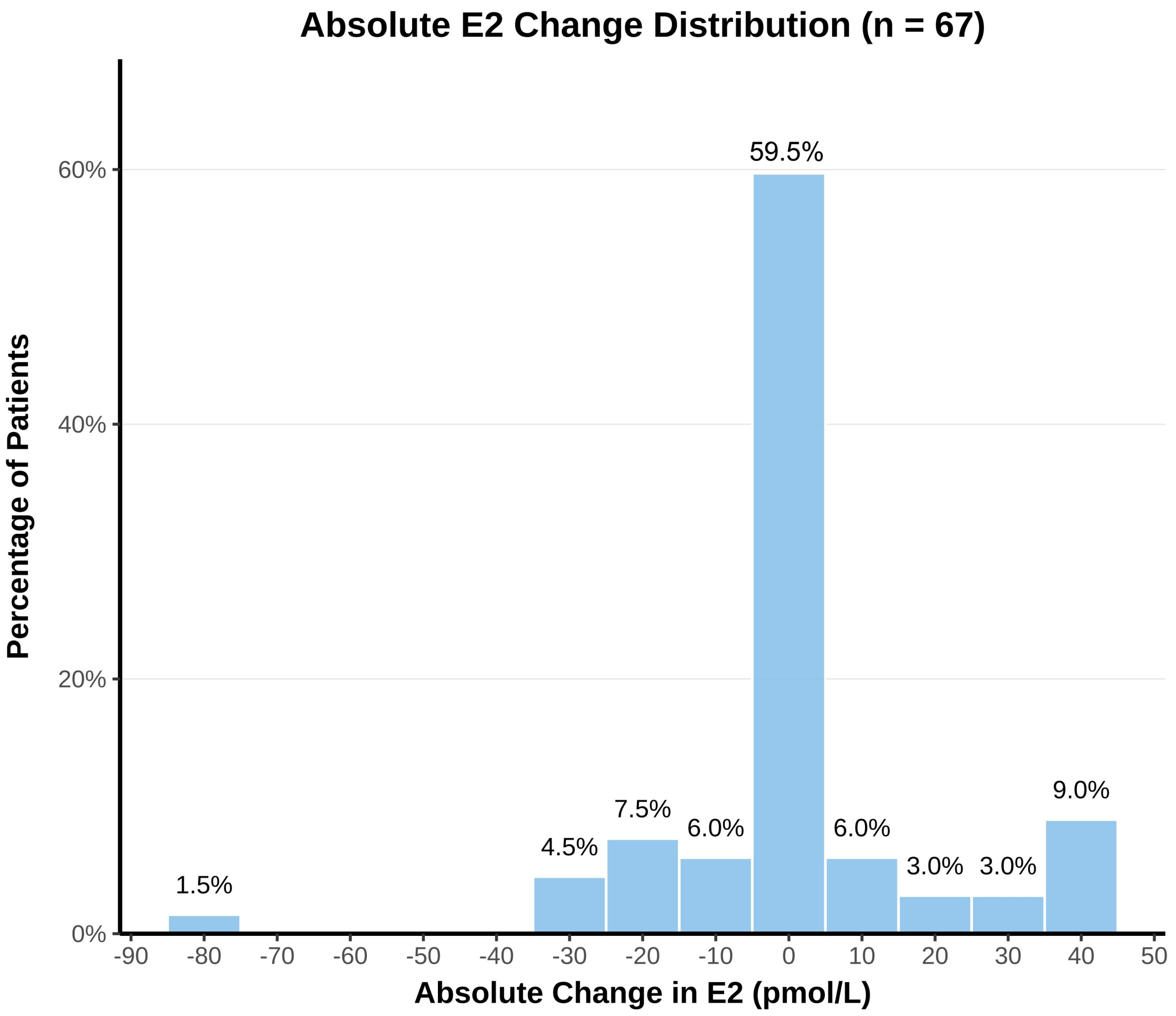

Histogram showing the distribution of the change in Estradiol concentration between the baseline and midterm measurement in the subset of patients (n = 67) who revisited for midterm measurements a median of 21.65 months (95% CI 19.46-23.84) after the baseline screening.E2 = Estradiol.

Supplemental Figure 7. Standardized change over time in Estradiol

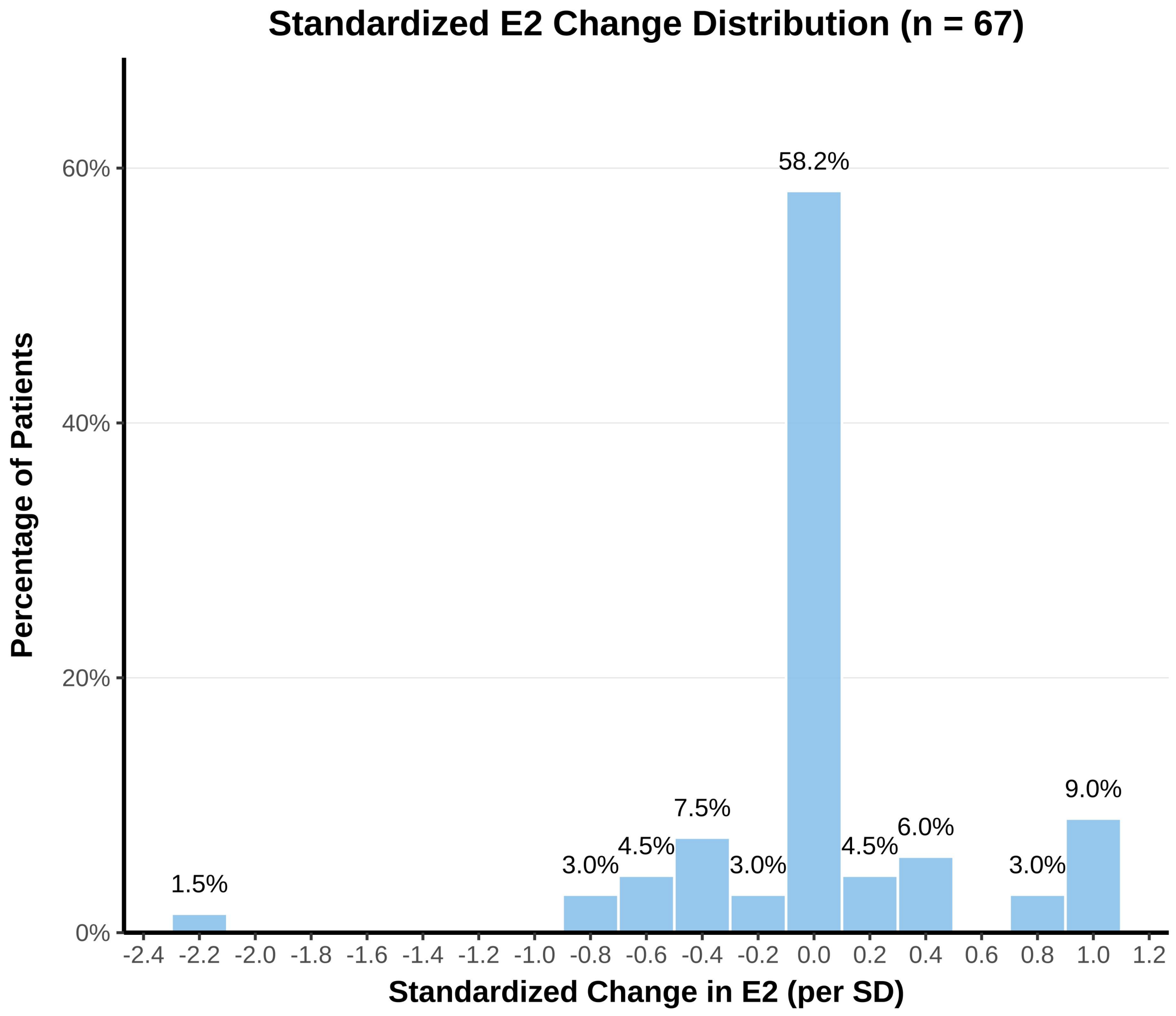

Histograms showing the distribution of the standardized change (i.e. change divided by the SD of the baseline measurement) between the baseline and midterm measurement of Estradiol in the subset of patients (n = 87) who revisited for midterm measurements a median of 21.65 months (95% CI 19.46-23.84) after the baseline screening. Estradiol, midterm visit, was not available for 20 patients, so for Estradiol the analysis was limited to the 67 patients with available measurements at both time points. E2 = Estradiol.

Supplemental Figure 8. Age-Specific Estrogen Dynamics in TS patients

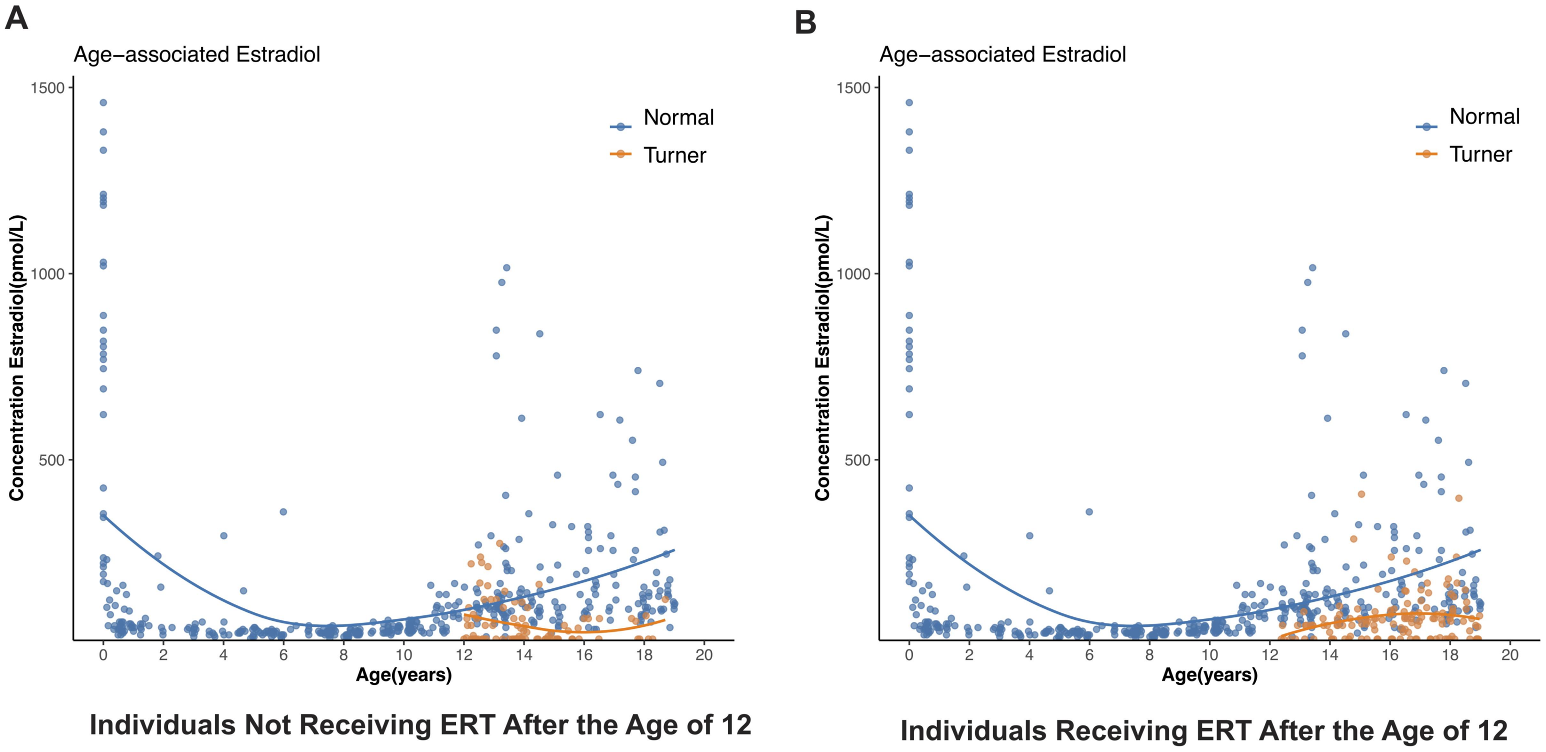

Serum estradiol levels in Turner syndrome (TS) patients versus age-matched healthy controls (longitudinal cohort analysis). Control data sourced from the CALIPER Pediatric Reference Interval Database; A. Estradiol deficiency in untreated TS girls >12 years relative to age-matched controls; B.Post-pubertal TS patients (>12 years) receiving ERT exhibit significantly lower estradiol levels than healthy peers (P<0.001).

Supplemental Figure 9. Association Between PTA and Sex Hormones

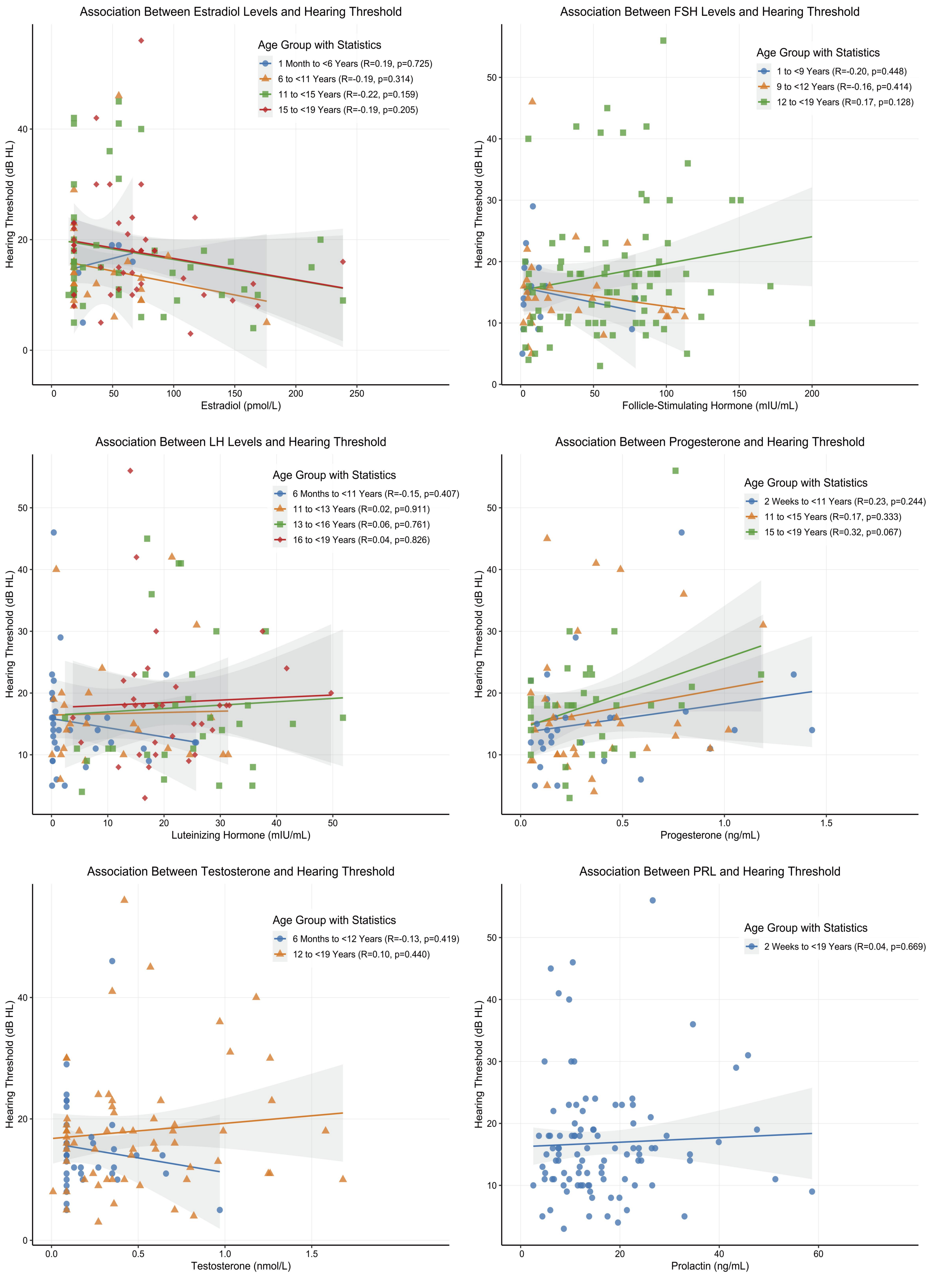

Scatterplots illustrate the associations between estradiol levels and pure-tone average (PTA) thresholds using longitudinal observations. The R value represents the Spearman rank correlation coefficient calculated across all data points, while the solid lines and shaded areas indicate the linear regression fit with 95% confidence intervals for each age group. P values denote the significance of the correlations. Age-specific stratification was conducted in alignment with CALIPER-defined reference intervals(https://caliper.research.sickkids.ca).
